# Proteomic and serologic assessments of responses to mRNA-1273 and BNT162b2 vaccines in human recipient sera

**DOI:** 10.3389/fimmu.2024.1502458

**Published:** 2025-01-27

**Authors:** Thomas E. Hickey, Uma Mudunuri, Heidi A. Hempel, Troy J. Kemp, Nancy V. Roche, Keyur Talsania, Brian A. Sellers, James M. Cherry, Ligia A. Pinto

**Affiliations:** ^1^ Vaccine, Immunity and Cancer Directorate, Frederick National Laboratory for Cancer Research, Frederick, MD, United States; ^2^ Advanced Biomedical Computational Science, Frederick National Laboratory for Cancer Research, Frederick, MD, United States; ^3^ Center for Human Immunology, Inflammation and Autoimmunity, National Institute of Allergy and Infectious Diseases, National Institutes of Health, Bethesda, MD, United States

**Keywords:** serology, proteomics, SARS-CoV-2, mRNA-1273, BNT162b2, vaccine response

## Abstract

**Introduction:**

The first vaccines approved against SARS-CoV-2, mRNA-1273 and BNT162b2, utilized mRNA platforms. However, little is known about the proteomic markers and pathways associated with host immune responses to mRNA vaccination. In this proof-of-concept study, sera from male and female vaccine recipients were evaluated for proteomic and immunologic responses 1-month and 6-months following homologous third vaccination.

**Methods:**

An aptamer-based (7,289 marker) proteomic assay coupled with traditional serology was leveraged to generate a comprehensive evaluation of systemic responsiveness in 64 and 68 healthy recipients of mRNA-1273 and BNT162b2 vaccines, respectively.

**Results:**

Sera from female recipients of mRNA-1273 showed upregulated indicators of inflammatory and immunological responses at 1-month post-third vaccination, and sera from female recipients of BNT162b2 demonstrated upregulated negative regulators of RNA sensors at 1-month. Sera from male recipients of mRNA-1273 showed no significant upregulation of pathways at 1-month post-third vaccination, though there were multiple significantly upregulated proteomic markers. Sera from male recipients of BNT162b2 demonstrated upregulated markers of immune response to doublestranded RNA and cell-cycle G(2)/M transition at 1-month. Random Forest analysis of proteomic data from pre-third-dose sera identified 85 markers used to develop a model predictive of robust or weaker IgG responses and antibody levels to SARS-CoV-2 spike protein at 6-months following boost; no specific markers were individually predictive of 6-month IgG response. Thirty markers that contributed most to the model were associated with complement cascade and activation; IL-17, TNFR pro-apoptotic, and PI3K signaling; and cell cycle progression.

**Discussion:**

These results demonstrate the utility of proteomics to evaluate correlates or predictors of serological responses to SARS-CoV-2 vaccination.

## 1 Introduction

The global response to the SARS-CoV-2 pandemic of 2020 ushered in a new chapter in vaccinology with the development and wide-spread use of mRNA-based vaccines (1). However, evidence is mounting that a more individualized approach may be needed as the COVID-19 landscape continues to evolve. The 2 original mRNA vaccines to SARS-CoV-2, BNT162b2 (Pfizer) and mRNA-1273 (Moderna) both target immune responses to full-length viral spike protein and have been highly successful in preventing severe COVID-19 disease, hospitalization, and death on the population level (2–5). However, the 2 vaccine types include different concentrations of mRNA per dose and have demonstrated differences in immunogenicity in different patient groups and between sexes assigned at birth (2–4, 6–10). In addition, multiple studies have shown that the circulating vaccine induced antibody levels decline rapidly in most recipients within a couple of months of primary series vaccination (7, 8, 11–13), and the Centers for Disease Control and Prevention (CDC) has since recommended additional vaccination doses and vaccines to develop sustainable and effective humoral responses (14). To add complexity, new viral variants routinely emerge and often escape neutralization, resulting in high numbers of breakthrough infections (15, 16). These observations support the CDC recommendation of booster immunizations for all recipients (17), however there are no known correlates of protection and it is not clear which patients will have robust versus weaker responses that may be strengthened through the administration of additional doses.

Established correlates of protection against SARS-CoV-2 infection or disease would be extremely valuable to inform recommendations for booster vaccinations. The mRNA-based SARS-CoV-2 vaccines are believed to impart protection through neutralizing antibody responses (as in other infections such as with Human Papillomavirus), as well as cell-mediated immune responses, and both responses result in distinct protein expression patterns (18–22). Proteomic changes in serum may consequently prove to be indicative, or even predictive, of vaccine immunogenicity and efficacy, and could inform new vaccine recommendations and developments.

The recent evolution of proteomic affinity-capture platforms into large comprehensive screening tools (23) provide a unique opportunity to evaluate broad spectrum protein responses in easily accessible, small volume serum samples (24). However, it remains to be demonstrated whether protein markers can be realistically used to predict serological immune responses. In this proof-of-concept study, a 7,289-proteome assay was used to evaluate human protein markers pre- and post-homologous third dose of mRNA-1273 or BNT162b2 in healthy vaccine recipients and then the results were compared with humoral response. Specifically, serum antibodies to SARS-CoV-2 spike, proteins, and impacted cellular pathways were analyzed in samples collected 1-month and 6-months after a third dose of vaccine and compared to pre-third dose samples. Influence of vaccine type and sex assigned at birth were also evaluated for differing antibody and proteomic profiles. In addition, pre-third dose sera were evaluated for proteomic markers that could be predictors of either higher or lower vaccine-induced serum IgG antibody content and used to develop a model predictive of 6-month antibody levels.

## 2 Materials and methods

### 2.1 Samples

Serum samples were collected from healthy vaccine recipients by Feinstein-Northwell Institute for Medical Research, Manhasset, NY (Institutional Review Board #20-1007) and by the National Institutes of Health’s Occupational Safety and Health Office located at Ft. Detrick, MD under the Research Donor Protocol (RDP). RDP participants were healthy NCI-Frederick employees and other NIH staff that donated blood samples for *in-vitro* research at the NCI-Frederick laboratories. The protocol is listed under NIH protocol number OH99CN046 and NCT number NCT00339911. Blood donors ranged in age from 25-76 years and included 67 females and 65 males; demographics are presented in **Table 1**. Study participants were sampled 61-377 days after administration of the second dose of the same primary series vaccine (either BNT162b2 or mRNA-1273) serving as a pre-boost timepoint. Participants were then boosted with a homologous third dose, and then sampled at 1-month (15 to 45 days post-third vaccination) and at 6-months (165-195 days post-third vaccination). Blood samples were processed at the collection sites, sera were frozen and stored at -80°C and then shipped on dry ice to the NCI-Frederick Repository until requested for testing at the Vaccine, Immunity, and Cancer Directorate (VICD).

**Table 1:** Study demographics.

Study Demographics		
Participants	BNT162b2	mRNA-1273
Number (n)	68	64
Geometric mean Age (Years)	46.6	42.5
Age Min-Max (Years)	25-71	25-76
Female (percent)	34 (50)	33 (52)
Male (percent)	34 (50)	31 (48)

### 2.2 Serology

#### 2.2.1 Enzyme-linked immunosorbent assay

ELISA assays used to quantify human serum IgG antibodies to SARS-CoV-2 spike protein were performed at room temperature (RT) as follows: Maxisorp 96-well plates (Thermo-Scientific Cat# 439454) were coated with recombinant SARS-CoV-2 spike protein (SARS-CoV-2 S-2P (14-1213)-T4f-His6) sourced from the Protein Expression Laboratory (PEL) at Frederick National Laboratory for Cancer Research (FNLCR), (0.15 µg/mL in phosphate-buffered saline [PBS]). After coating for a minimum of 24 hours at 4°C, assay plates were washed with a PBS-Tween buffer and blocked with PBS-Tween 0.20% and 4.00% skim milk (BD, Cat# 232100) for 90 minutes. Following a plate wash, heat-inactivated samples were tested with appropriate in-well dilution series. Plates were incubated for 60 minutes with the samples, washed, and then incubated for an additional 60 minutes with an empirically determined dilution of goat anti-human IgG HRP-conjugate in PBS-Tween (Seracare, Cat# 5220-0390). The plates were washed and developed with tetramethylbenzidine (TMB) 2-component substrate (Seracare, Cat# 5120-0049, 5120-0038) for 25 minutes. Finally, the reaction in the plate wells was stopped with 0.36N sulfuric acid and read at 450nm and 620nm on a SpectraMax plate reader (Molecular Devices). Data analyses were performed using SoftMax Pro GxP 7.0.3. Reportable values for IgG quantitative ELISA are binding antibody units per milliliter (BAU/mL), based on a standard calibrated to the World Health Organization (WHO) International Standard (25).

#### 2.2.2 Avidity enzyme-linked immunosorbent assay (chaotrope ELISA)

Avidity ELISA assays (chaotrope assays) are based on standard ELISA tests for anti-SARS-CoV-2 spike protein IgG but include an additional step where the analyte (antibody) is exposed to a chaotropic agent that effectively breaks and elutes off weakly bound antibody species; a “bind and break” ELISA. Urea was used as the chaotropic agent due to its experimental range and minimal impact on assay plate coat integrity. Avidity ELISAs were performed on sample dilutions with optical densities (OD) between 0.50 to 1.30 OD units at 450nM; 1.00 was the target OD. Extensive assay development with multiple SARS-CoV-2 serum samples demonstrated highly reproducible (CV<10%) measurements in this range (13). Each assay plate tested 5 serum samples in duplicate. After each sample was incubated on the assay plate for 1 hour at 22°C (room temperature [RT]), the plates were washed and incubated with dilutions of urea ranging from 0 to 10M for 15 minutes at 22°C. After 4 washes with PBS-Tween, plates were developed as described above for the quantitative IgG assay. Serum avidity assessments are reported as Avidity Indices (AI20); the molar concentration (M) of chaotrope required to reduce the optical density of the sample to 80% that of untreated wells. Additionally, each assay plate contained 2 system suitability controls that were developed from well characterized serum samples: 1 control with a known low avidity index, the other a known high avidity index.

### 2.3 Proteomics

SomaLogic’s SomaScan v4.1 7K Assay platform was used to evaluate serum protein content longitudinally for significant changes in abundance at 1-month and 6-months following homologous third vaccination. Protein quantitated in sera collected pre-third dose set baseline values for each study participant. The SomaScan assay was performed on a Tecan Fluent 780 high throughput system according to manufacturer’s instructions at the NIH Center for Human Immunology. A complete list of the 7,289 targets analyzed is available at https://menu.somalogic.com. The data are reported in relative fluorescence units (RFU), a surrogate for protein concentration. The SomaScan Assay data were normalized using SomaLogic’s standardization procedure (26). In short, data were first normalized to correct for well-to-well variation in microarray hybridization steps. This was then followed by intraplate median signal normalization to correct for sample-to-sample differences that may be introduced due to technical assay effects. Plate scaling was applied to normalize global signal differences between plates, and calibration was applied to adjust for SOMAmer reagent-specific differences between tests. Finally, median signal normalization was performed via Adaptive Normalization by Maximum Likelihood (ANML) to harmonize data across multiple assay plates.

### 2.4 Data analyses

Serum IgG antibody to SARS-CoV-2 spike concentrations are reported as geometric mean binding antibody units per milliliter (BAU/mL) based on established serological WHO standards (27).

Proteomic data were stratified based on vaccine received and sex assigned at birth for these analyses. In total, data analyses were performed on 12 data subsets as follows: 2 vaccines assessed at 3 timepoints and against 2 sex assigned at birth stratifications.

### 2.5 Antibody and avidity level analyses

Serum antibody levels and avidity are expressed as geometric mean concentrations (GMC) and geometric mean avidity levels (GMA), respectively, with corresponding 95% confidence intervals. A Mann-Whitney U test (Wilcoxon Rank Sum test) was used when comparing antibody content or avidity values between vaccine type, or between sex assigned at birth. A Wilcoxon Signed Rank test was used when serological tests were evaluated between timepoints. These tests were chosen because they do not have the requirement of a normal distribution of values, as in an unpaired or paired t-test, and work for smaller sample sizes; p<0.05 was considered significant.

### 2.6 Proteomic data analysis

Serum proteins were quantitated using the SomaScan v4.1 7K Assay platform (28, 29). The geometric mean value for each detectable protein within each cohort was quantitated in relative fluorescent units (RFU), and the magnitude of protein abundance change was expressed with the following formulas: log2(1-month value/pre-third-dose value) and log2(6-month value/pre-third-dose value). A 2-tailed t-test was performed to determine if the calculated geometric mean values were significantly different between the 2 timepoints. p-values in this assessment provided significance thresholds. Proteins having a p-value of <0.05 and a log protein abundance difference of >0.20 (significant increase) or <-0.20 (significant decrease) were identified as proteins that demonstrated significant differences in content.

### 2.7 Pathway analyses

Biological processes impacted by vaccination were evaluated through KEGG and REACTOME pathway functional enrichment analyses. Enrichment ratio and false discovery rate (FDR) were calculated for significant proteins using the WEB-based GEne SeT AnaLysis Toolkit (WebGestalt) 2 (30). EntrezGene was used to map proteins to gene identifiers. Background gene sets for enrichment analyses were created by selecting all unique EntrezGene names from all protein analytes detected in the SomaScan Assay.

### 2.8 Predictive analyses

As serological antibody responses to the 2 vaccines were largely analogous irrespective of vaccine or sex assigned at birth (**Tables 2**, **3**), and because of the limited sizes of the vaccine recipient cohorts, the entire database was evaluated without segregation by sex assigned at birth or vaccine to identify pre-third-dose markers predicting 6-month antibody response levels. Attempts to develop predictive models specific for sex assigned at birth or vaccine were unsuccessful lacking sufficient predictive accuracy; larger sample sizes would be necessary to develop segregated models.

**Table 2:** Serology responses to vaccination, influence of vaccine type.

Response to Homologous third Vaccination			
Geometric mean IgG Content, 95% CI:(BAU/mL)			
	Pre-third vaccination	1-month	6-months
mRNA-1273	619 (427-896)	8655 (7074-10591)	3074 (2382-3966)
BNT162b2	268 (212-340)	7635 (6542-8910)	2823 (2222-3587)
p-value	0.0006	0.3008	0.5872
Avidity Development (M)			
	Pre-third vaccination	1-month	6-months
mRNA-1273	4.1 (3.8-4.3)	5.5 (5.4-5.6)	5.3 (5.1-5.5)
BNT162b2	3.5 (3.3-3.8)	5.4 (5.2-5.5)	5.3 (5.1-5.5)
p-value	0.0247	0.3640	0.8964

**Table 3:** Circulating IgG antibody content to SARS-CoV-2 Spike, Influence of sex assigned at birth.

Influence of sex assigned at birth on Response to Homologous third Vaccination Geometric mean IgG Content, 95% CI:(BAU/mL)			
	Pre-third vaccination	1-month	6-months
Male	424 (298-602)	8124 (6757-9768)	3175 (2423-4161)
Female	383 (285-513)	8088 (6804-9614)	2747 (2193-3440)
p-value	0.6273	0.7382	0.6434
Male Vaccine Recipients Geometric mean IgG Content, 95% CI:(BAU/mL)						
	Pre-third vaccination	1-month	6-months
mRNA-1273	688 (391-1214)	9400 (6936-12739)	3879 (2570-5855)
BNT162b2	272 (183-406)	7173 (5707-9016)	2682 (1856-3875)
p-value	0.0092	0.1940	0.2403
Female Vaccine Recipients Geometric mean IgG Content, 95% CI:(BAU/mL)						
	Pre-third vaccination	1-month	6-months
mRNA-1273	560 (336-932)	8050 (6070-10675)	2540 (1839-3510)
BNT162b2	265 (202-347)	8126 (6535-10103)	2963 (2133-4115)
p-value	0.0436	0.9851	0.6582

**Figure 1:**
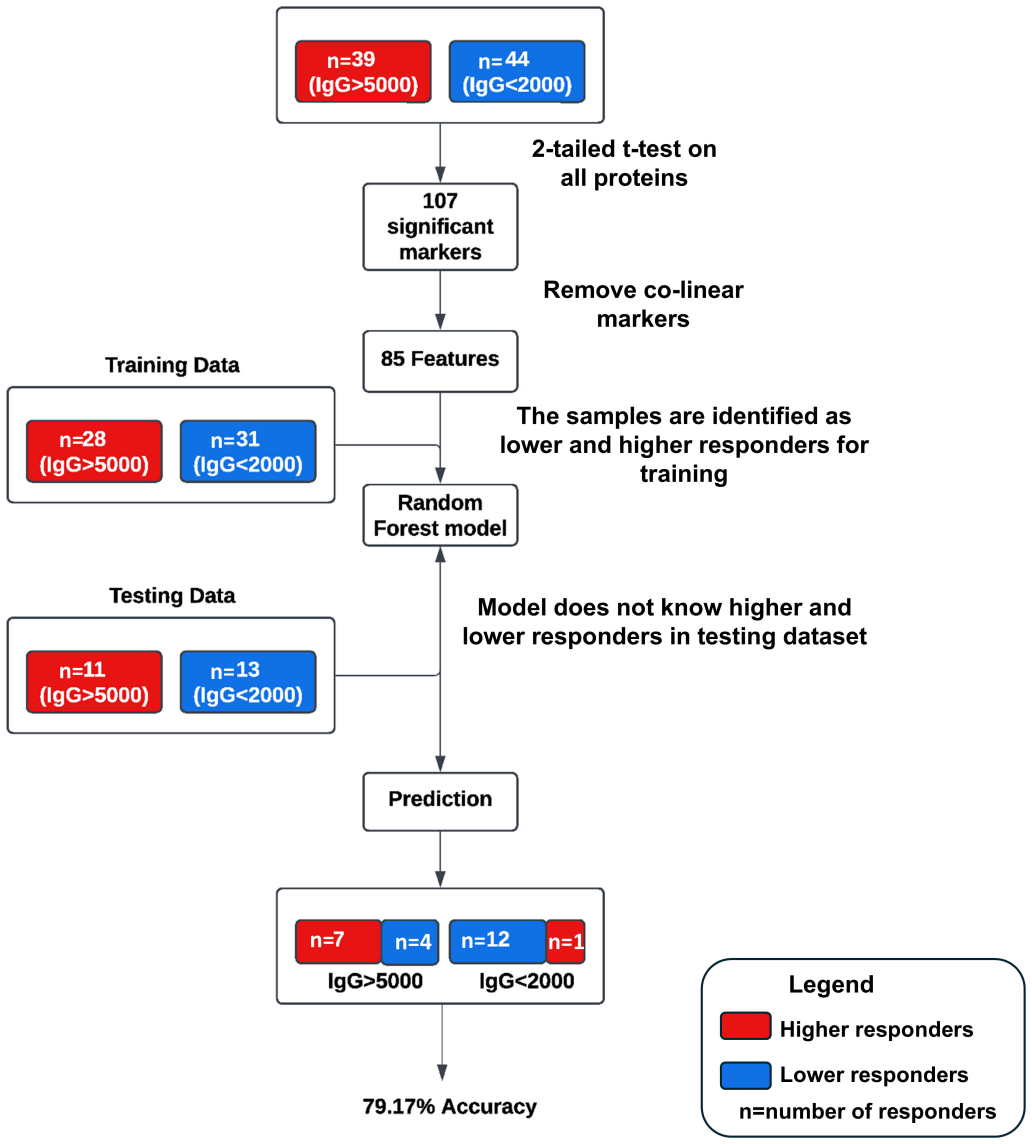
Schematic of the pilot machine learning model. Thirty-nine subjects were identified as higher responders (IgG > 5000 BAU/mL) and 44 subjects were identified as lower responders (IgG < 2000 BAU/mL) based on IgG levels at 6-months. One hundred and seven statistically significant markers were identified based on comparative changes between the extreme (higher or lower) responders (p<0.05). The markers were reduced to an 85-feature set by removing redundant predictors, or co-linear markers. The RF model was trained with data from 28 higher responders and 31 lower responders, and the model was tested on the remaining 11 higher responders and 13 lower responders and achieved a predictive accuracy of 79.17% on test data with an area under the curve (AUC) of 86.71%.

A pilot machine learning model was developed (**Figure 1**) using Random Forest (RF) to predict antibody response levels at 6-months. Thirty-nine subjects were identified as higher responders (IgG > 5000 BAU/mL) and 44 subjects were identified as lower responders (IgG < 2000 BAU/mL) based on IgG levels at 6-months. A 2-tailed t-test was performed to determine if the calculated geometric mean values of the protein levels at pre-third dose for the 2 groups were significantly different from zero. One hundred and seven statistically significant markers were identified based on comparative changes between the extreme (higher or lower) responders (p<0.05). The markers were reduced to an 85-feature set by removing redundant predictors, or co-linear markers. The RF model was trained with data from 28 higher responders and 31 lower responders, and the model was tested on the remaining 11 higher responders and 13 lower responders and achieved a predictive accuracy of 79.17% on test data with an area under the curve (AUC) of 86.71%.

### 2.9 Confounding factors to predictive model development

Assessments of vaccination responses were confounded with the observation that 18 mRNA-1273 and 19 BNT162b2 recipients had antibody reactive to SARS-CoV-2 nucleocapsid protein, despite no self-reports of infection indicating coronavirus infections or potential exposure. Moreover, 14 mRNA-1273 and 7 BNT162b2 vaccine recipients had positive nucleocapsid tests at 1-month and 6-month timepoints, possibly indicating subclinical infections. To evaluate the priming influence of the infections, the dataset was re-examined excluding vaccine-recipient samples that tested positive for coronavirus nucleocapsid. Evaluation of nucleocapsid-antibody negative sera did not significantly affect pre-third-dose predictive marker sets or pathways associated with higher or lower 6-month serology. Analyses of the nucleocapsid naïve dataset did produce 1 additional cellular process - mitophagy (selective degradation of defective mitochondria) -which contributed to model development (Data not shown).

## 3 Results

### 3.1 There were no significant serological differences based on vaccine-received or sex assigned at birth

**Figure 2:**
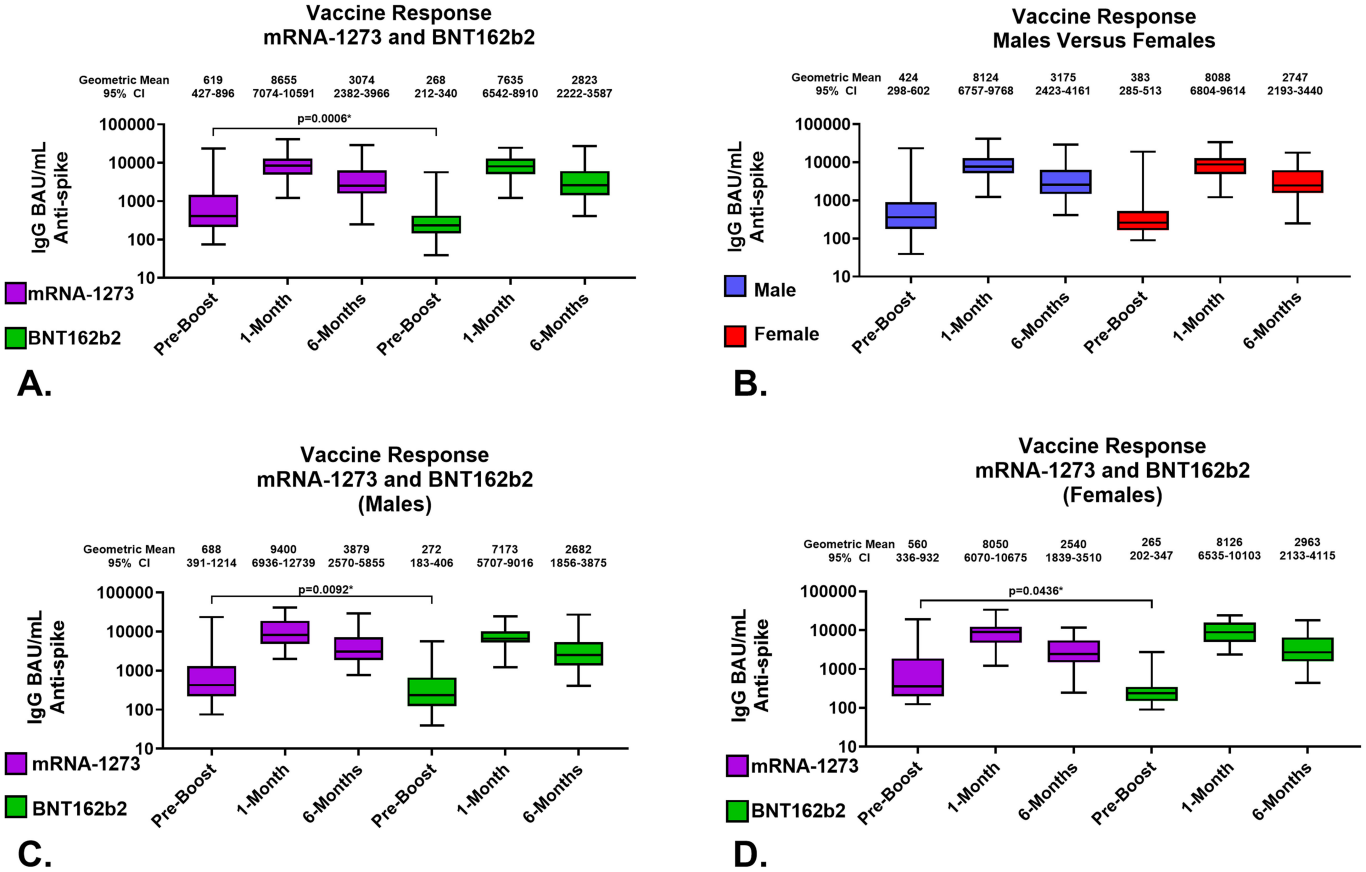
Serological Assessments of Responses to Homologous Third Vaccination. Assessments of IgG anti-SARS-CoV-2 Spike content of serum. **(A)** Comparison of serum antibody levels pre-boost and 1 and 6 months after vaccination. **(B)** Response to third vaccination by sex assigned at birth; male recipients (Blue), female recipients (Red). **(C)** Vaccine response to homologous third vaccination in sera from male recipients. **(D)** Vaccine response to homologous third vaccination in sera from female recipients. mRNA-1273 results are shown in purple, BNT162b2 in green. Group geometric mean vales are depicted by the bar graph horizontal line and listed above each bar with the 95 percent confidence interval of the geometric mean. Statistically significant comparisons are depicted with p values.

The development of a predictive model of 6-month IgG antibody response levels from vaccination through proteomic assessments of sera required desegregated analyses due to study size. Consequently, we evaluated in-depth both the quality and robustness of the IgG responses by vaccine received and sex assigned at birth to assure validity of our desegregated assessment. Serological responses were comparable across both vaccine types and sexes assigned at birth at both 1-month and 6-months post-third vaccinations (**Figures 2A, B**; **Tables 2**, **3**); the only significant differences were detected in pre-third-dose sera (mRNA-1273 primary vaccine recipients demonstrated statistically higher antibody and avidity levels to spike compared to recipients of BNT162b2, irrespective of sex assigned at birth [antibody levels: p=0.0006 vaccines, p=0.0092 males, p=0.0436 females; avidity: p=0.0247]) (**Figures 2A, C, D**; **Table 2**). Additionally, the mean time between primary series vaccination and third dose of vaccine was different in the 2 vaccine groups: 294 days (164-377) for mRNA-1273 recipients and 265 days (61-377) for BNT162b2 recipients (p<0.0001, Mann Whitney).

In detail, the geometric mean anti-SARS-CoV-2 spike IgG antibody levels in mRNA-1273 and BNT162b2 recipients at 1-month were 8655 (95% CI: 7074-10591) BAU/mL and 7635 (95% CI: 6542-8910) BAU/mL, respectively; p=0.3008 (**Figure 2A**; **Table 2**). The geometric mean antibody avidity levels at 1-month for mRNA-1273 and recipients BNT162b2 were 5.5 (95% CI: 5.4-5.6) M and 5.4 (95% CI: 5.2-5.5) M, respectively; p=0.3640 (**Table 2**). The 6-month geometric mean anti-SARS-CoV-2 levels for mRNA-1273 and BNT162b2 recipient sera were 3074 (95% CI: 2382-3966) BAU/mL and 2823 (95% CI: 2222-3587) BAU/mL, respectively; p=0.5872 (**Figure 2A**; **Table 2**). The geometric mean antibody avidity levels at 6-months for mRNA-1273 and BNT162b2 recipients were 5.3 (95% CI: 5.1-5.5) M and 5.3 (95% CI: 5.1-5.5) M, respectively; p=0.8964 (**Table 2**).

When serology responses were analyzed according to sex assigned at birth, male and female recipients had geometric mean serum antibody levels at 1-month of 8124 (95% CI: 6757-9768) BAU/mL and 8088 (95% CI: 6804-9614) BAU/mL, respectively; p=0.7382 (**Figure 2B**; **Table 3**). At 6-months, male and female recipients had geometric mean serum antibody levels of 3175 (95% CI: 2423-4161) BAU/mL and 2747 (95% CI: 2193-3440) BAU/mL, respectively; p=0.6434 (**Figure 2B**; **Table 3**). There were also no statistically significant serum avidity differences based on sex assigned at birth (data not shown).

Further analyzing according to both sex assigned at birth and vaccine, male recipients of mRNA-1273 and BNT162b2 at 1-month demonstrated geometric mean serum antibody levels of 9400 (95% CI: 6936-12739) BAU/mL and 7173 (95% CI: 5707-9016) BAU/mL, respectively; p=0.1940 (**Figure 2C**; **Table 3**). Female recipients of mRNA-1273 and BNT162b2 at 1-month had geometric mean serum antibody levels of 8050 (95% CI: 6070-10675) BAU/mL and 8126 (95% CI: 6535-10103) BAU/mL, respectively; p=0.9851 (**Figure 2D**; **Table 3**). At 6-months, male recipients of mRNA-1273 and BNT162b2 had geometric mean serum antibody levels of 3879 (95% CI: 2570-5855) BAU/mL and 2682 (95% CI: 1856-3875) BAU/mL, respectively; p=0.2403 (**Figure 2C**; **Table 3**). Finally, at 6-months female recipients of mRNA-1273 and BNT162b2 had geometric mean serum antibody levels of 2540 (95% CI: 1839-3510) BAU/mL and 2963 (95% CI: 2133-4115) BAU/mL, respectively; p=0.6582 (**Figure 2D**; **Table 3**).

### 3.2 Proteomic Assessments of cohorts by vaccine and sex assigned at birth

**Figure 3:**
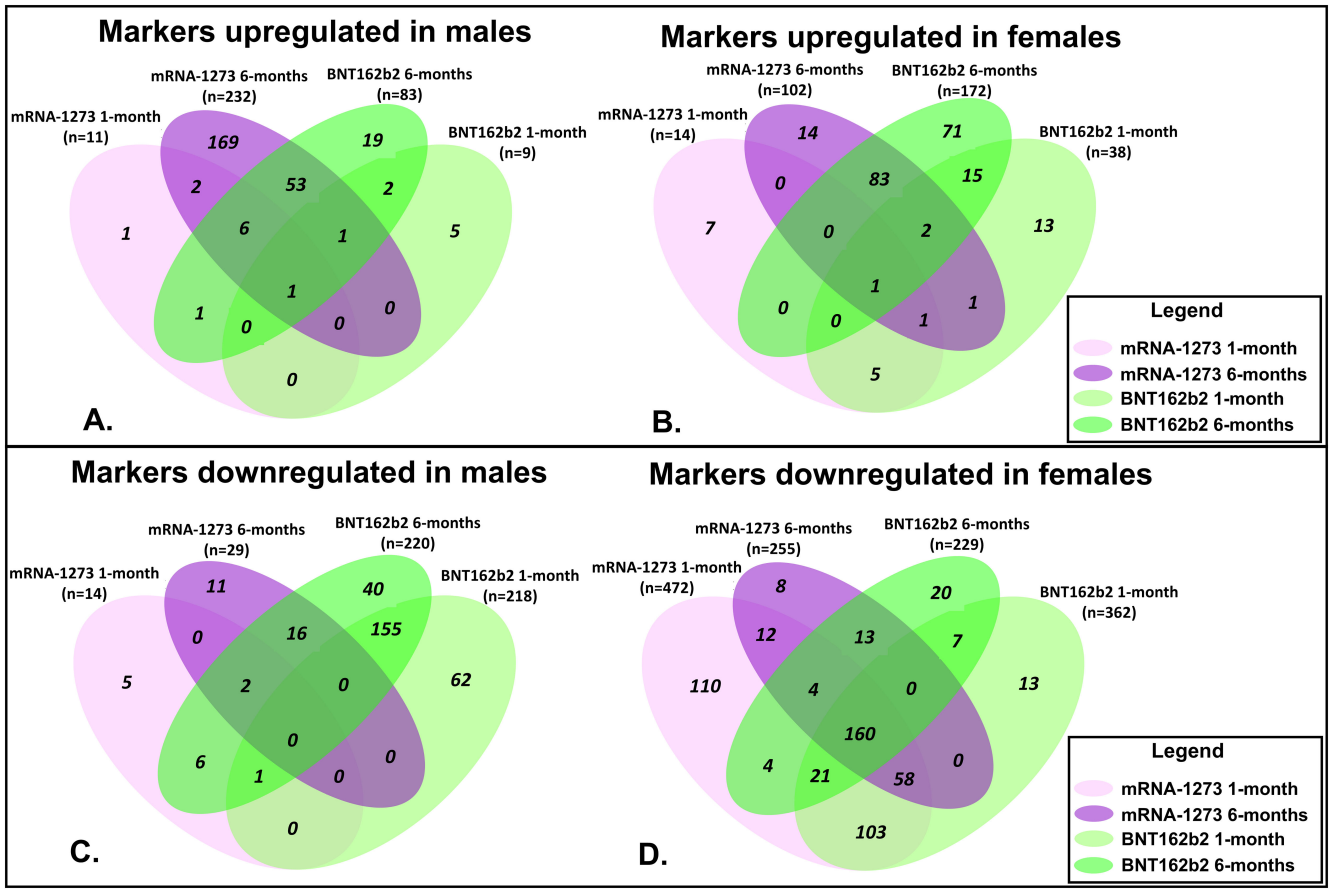
Assessment of Proteomic Marker Profiles by Vaccination Cohort and sex assigned at birth. Assessment of proteomic marker changes from baseline measurement. **(A, B)** are diagrams of assessment of markers upregulated at 1-month and 6-months in sera from male and female recipients (respectively); **(C, D)** are diagrams of assessment of markers downregulated at 1- and 6-months in sera from male and female recipients (respectively). mRNA-1273 vaccinated (1-month) light purple and (6-month) dark purple. BNT162b2 vaccinated (1-month) light green and (6-month) dark green.

**Figure 4:**
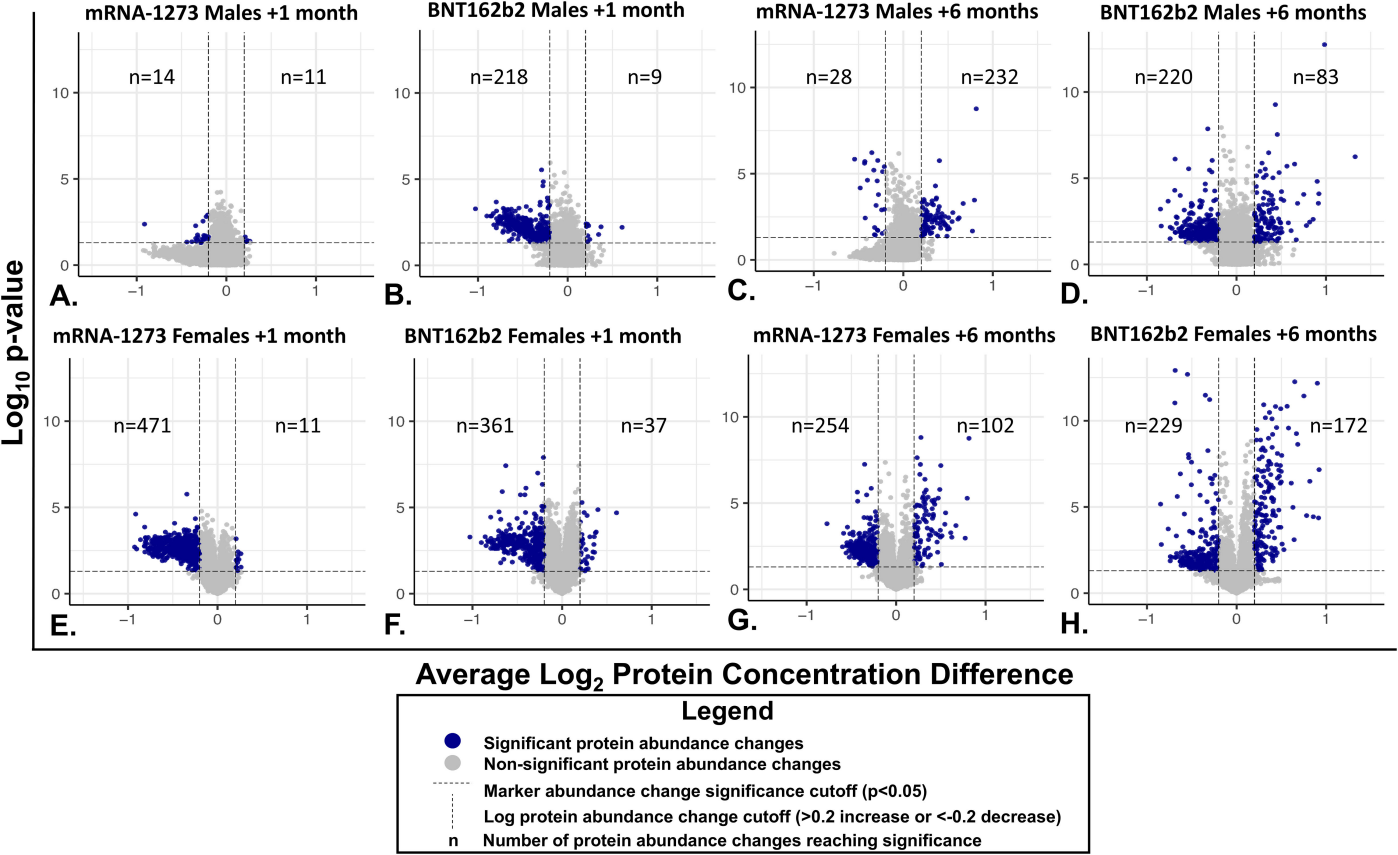
Individual Serum Protein Abundance Change and Statistical Significance at 1-month and 6-Months After Third Vaccination. Each graphed point represents a serum protein marker with detectable change from pre-vaccination levels. Statistical significance (P<0.05) is depicted on the y-axes and Log2 abundance change on the X-axes. Lines of significance (p<0.05 and abundance change <0.2 or >0.2 m) are depicted by dashed lines. Responses in sera from male recipients are shown in panels **(A–D)**, responses in serum from female recipients are shown in the bottom panels **(E–H)**. **(A, B, E, F)** Responses 1 month after vaccination. **(C, D, G, H)** 6-month responses. Significant marker changes are depicted with blue dots; grey dots depict marker abundance changes that did not reach significance. N values are listed in each quadrant for significant marker changes.

Proteomic profiles in serum from each vaccine recipient were assessed with the SomaScan v4.1 7K Assay platform before the third vaccination (after completion of 2-dose primary vaccine series), and then at 1-month and 6-months post-third vaccination; summary of changes in protein marker expression profiles of the respective cohorts are presented in **Supplementary Table S1A** (downregulated markers) and **Supplementary Table S1B** (upregulated markers) and **Figure 3**. Significant proteins were selected based on p-value and average log abundance differences (**Figure 4**; **Supplementary Tables S1A, B**).

Sera collected from the male recipients of mRNA-1273 1 month post-third vaccination demonstrated upregulation of 11 markers and downregulation of 14, while sera from female recipients showed upregulation of 14 markers and downregulation of 472 (**Figure 3**; **Supplementary Tables S1A, B**). By 6 months, sera from the male recipients of mRNA-1273 showed upregulated 232 protein markers and downregulated 29, while the sera from the female recipient group demonstrated upregulated 102 markers and downregulated 255 (**Figure 3**; **Supplementary Tables S1A, B**).

The sera from the male recipients of BNT162b2 showed upregulation of 9 markers at 1-month and downregulation of 218 markers post-third vaccination, while sera from the female recipient cohort demonstrated upregulated 38 markers and downregulated 362 proteins (**Figure 3**; **Supplementary Tables S1A, B**). At 6-months post-third vaccination, sera from male recipients showed upregulated 83 markers and downregulated 220, while sera from female recipients demonstrated upregulated 172 markers and downregulated 229 (**Figure 3**; **Supplementary Tables S1A, B**).

When evaluated at the cohort level, sera from male recipients of either vaccine at 1-month post-third vaccination demonstrated 1 common upregulated marker (UB2D1/PolyUbiquitin K48), and 1 common downregulated marker (CXCL8, interleukin-8) (**Figure 3**). In sera from the female recipient cohorts, 7 markers were upregulated (UBE2D1|UBB, CHAC1, LEP, CST5, CST2, INS) and 342 markers downregulated (**Figure 3**). By 6-months, sera from male recipients of either vaccine upregulated 61 markers and downregulated 18, while sera from female recipients of either vaccine demonstrated upregulation of 86 markers and downregulation of 177 (**Figure 3**; **Table 4**; **Supplementary Tables S1A, B**). Upregulation of UB2D1/PolyUbiquitin K48 was common to sera from all recipient cohorts at 1-month after third vaccination, regardless of sex assigned at birth or vaccine received (**Figure 3**).

**Table 4:** Top 10 markers upregulated 1-month post-third vaccination.

Upregulated mRNA-1273 Female Recipients (1-month)			
Name	Marker	Abundance (RFU)	Significance (p)
UB2D1/PolyUbiquitin K48	UBE2D1|UBB	0.262310471	0.028873413
No protein		0.260175482	0.004812444
Cystatin-S	CST4	0.238394992	0.010329505
Melittin.VESMG	MELT	0.234682716	0.012103636
D-dimer	FGA|FGB|FGG	0.229641179	0.003727817
Cystatin-D	CST5	0.226042814	0.00631462
Band 4.1-like protein 1	EPB41L1	0.224335353	0.046323173
Insulin	INS	0.222642643	0.015592254
Histatin-3	HTN3	0.220599779	0.010398185
Glutathione-specific gamma-glutamylcyclotransferase 1	CHAC1	0.210679991	0.028781324
Upregulated mRNA-1273 Male Recipients (1-month)			
Name	Marker	Abundance (RFU)	Significance (p)
Porphobilinogen deaminase	HMBS	0.386411533	0.037515731
Tubulin-specific chaperone cofactor E-like protein	TBCEL	0.331158567	0.044087199
Band 4.1-like protein 1	EPB41L1	0.322041671	0.042732449
Eukaryotic translation initiation factor 2C 2	AGO2	0.318949382	0.049328952
Flavin reductase (NADPH)	BLVRB	0.317039757	0.03549251
UB2D1/PolyUbiquitin K48	UBE2D1|UBB	0.312316601	0.040253001
Platelet-activating factor acetylhydrolase IB subunit gamma	PAFAH1B3	0.311850831	0.034457691
Ubiquitin carboxyl-terminal hydrolase 14	USP14	0.310442854	0.025334095
Tropomodulin-1	TMOD1	0.284304196	0.029963826
Insulin	INS	0.230907349	0.043061194
Upregulated BNT162b2 Female Recipients (1-month)			
Name	Marker	Abundance (RFU)	Significance (p)
UB2D1/PolyUbiquitin K48	UBE2D1|UBB	0.607793151	2.04E-05
Lysosomal alpha-glucosidase	GAA	0.399913314	1.34E-05
Matrix Gla protein	MGP	0.380325892	0.000263212
Cancer/testis antigen 1	CTAG1A|CTAG1B	0.372971351	0.000292772
6-phosphogluconate dehydrogenase, decarboxylating	PGD	0.361802534	0.003981293
Glucagon	GCG	0.358596605	0.000498532
Proenkephalin-A	PENK	0.355051683	0.001395606
Glutathione-specific gamma-glutamylcyclotransferase 1	CHAC1	0.346030491	0.003767481
Poly(rC)-binding protein 2	PCBP2	0.335035501	0.008597658
Metalloproteinase inhibitor 3	TIMP3	0.310196296	0.000501893
Upregulated BNT162b2 Male Recipients (1-month)			
Name	Marker	Abundance (RFU)	Significance (p)
UB2D1/PolyUbiquitin K48	UBE2D1|UBB	0.319356416	0.006160708
Alcohol dehydrogenase 1C	ADH1C	0.28002833	0.019782399
Glutathione-specific gamma-glutamylcyclotransferase 1	CHAC1	0.278445535	0.016013345
Cytochrome P450 2C19	CYP2C19	0.260331428	0.028872966
Cancer/testis antigen 1	CTAG1A|CTAG1B	0.244254885	0.005766592
Alcohol dehydrogenase 4	ADH4	0.240887034	0.024632752
Sorbitol dehydrogenase	SORD	0.227359432	0.017601475
Formimidoyltransferase-cyclodeaminase	FTCD	0.216772387	0.010574406
Apolipoprotein A-V	APOA5	0.208358558	0.002681697

Sorted for p values first, then largest (abundance) change.

Evaluation of the 10 most significant (highest statistical significance/abundance change) proteomic markers upregulated in sera at 1-month after the third dose of vaccine demonstrated that both vaccine groups modulated sets of vaccine-type associated markers, regardless of sex assigned at birth. Specifically, sera from both mRNA-1273 recipient cohorts showed upregulated UB2D1/PolyUbiquitin K48 (UBE2D1|UBB), Insulin (INS) and Band 4.1-like protein1 (EPB41L1), while sera from both BNT162b2 recipient cohorts showed upregulated UB2D1/PolyUbiquitin K48 (UBE2D1|UBB), Glutathione-specific gamma-glutamylcyclotransferase 1 (CHAC1), and Cancer/testis antigen 1 (CTAG1A|CTAG1B) (**Table 4**).

### 3.3 Proteomics assessments of biological pathways and processes

Biological pathways were assessed based on observed proteomic marker changes according to vaccine received and sex assigned at birth. Proteins with significant changes between pre-third dose and 1-month or 6-months after third dose of vaccine were assessed through REACTOME and KEGG databases to evaluate differential pathways and cellular processes impacted (31, 32). A complete accounting at the cohort level of pathways up- and downregulated in 6-month sera compared to pre-third-dose sera measurements are listed in **Table 5**.

**Table 5:** Cellular pathways and processes impacted by third vaccination.

Upregulated Pathways			
1-month			
Cohort	Pathway	Enrichment Ratio	FDR
mRNA-1273 Female Recipients	Amyloid fiber formation	30.328125	0.01268247
	Common Pathway of Fibrin Clot Formation	48.525	0.00558661
	Formation of Fibrin Clot (Clotting Cascade)	27.7285714	0.01468566
	GRB2:SOS provides linkage to MAPK signaling for Integrins	80.875	0.00291728
	p130Cas linkage to MAPK signaling for integrins	34.6607143	0.00973617
	Regulation of TLR by endogenous ligand	64.7	0.00360031
	Salivary secretion	44.6206897	0.00223322
	Toll-like Receptor Cascades	16.0148515	0.00360031
	MAP2K and MAPK activation	34.6607143	0.00973617
	Oncogenic MAPK signaling	26.2297297	0.01640566
	Paradoxical activation of RAF signaling by kinase inactive BRAF	35.9444444	0.00943464
	Platelet Aggregation (Plug Formation)	35.9444444	0.00943464
	Signaling by BRAF and RAF fusions	28.5441177	0.01429216
	Signaling by high-kinase activity BRAF mutants	38.82	0.00943464
	Signaling by moderate kinase activity BRAF mutants	35.9444444	0.00943464
	Signaling by RAS mutants	33.4655172	0.01006548
	Synthesis, secretion, and deacylation of Ghrelin	53.9166667	0.0471068
6-month			
Cohort	Pathway	Enrichment Ratio	FDR
mRNA-1273 Female Recipients	Cap-dependent Translation Initiation	9.55485232	0.0020531
	Eukaryotic Translation Elongation	14.3322785	3.43E-04
	Eukaryotic Translation Initiation	9.55485232	0.0020531
	Eukaryotic Translation Termination	14.955421	3.19E-04
	Formation of a pool of free 40S subunits	13.7589873	3.78E-04
	GTP hydrolysis and joining of the 60S ribosomal subunit	10.1169025	0.00158428
	Infectious disease	4.89394875	2.17E-04
	Influenza Infection	10.1918425	2.17E-04
	Influenza Life Cycle	10.289841	3.96E-04
	Influenza Viral RNA Transcription and Replication	11.4658228	9.45E-04
	Eukaryotic Translation Termination	14.955421	3.19E-04
	L13a-mediated translational silencing of Ceruloplasmin expression	10.4234753	0.00144894
	Major pathway of rRNA processing in the nucleolus and cytosol	9.29661307	3.51E-05
	Metabolism of RNA	4.45020519	2.17E-04
	mRNA Splicing	7.37088608	3.43E-04
	mRNA Splicing - Major Pathway	7.16613924	3.78E-04
	Nonsense Mediated Decay (NMD) enhanced by the Exon Junction Complex (EJC)	13.838062	2.17E-04
	Nonsense Mediated Decay (NMD) independent of the Exon Junction Complex (EJC)	14.3322785	3.43E-04
	Nonsense-Mediated Decay (NMD)	13.838062	2.17E-04
	Peptide chain elongation	16.3797468	2.17E-04
	Processing of Capped Intron-Containing Pre-mRNA	6.99135536	2.17E-04
	Regulation of expression of SLITs and ROBOs	7.05589094	0.00116369
	Ribosome	11.0959575	0.0010946
	rRNA processing	7.64388186	0.00653237
	rRNA processing in the nucleus and cytosol	8.38962643	0.00394799
	Selenoamino acid metabolism	10.1169025	0.00158428
	Selenocysteine synthesis	16.3797468	2.17E-04
	Signaling by ROBO receptors	4.77742616	0.01318081
	Spliceosome	7.29643268	0.0024757
	SRP-dependent cotranslational protein targeting to membrane	11.4658228	9.45E-04
	Viral mRNA Translation	17.1987342	2.17E-04
1-month			
Cohort	Pathway	Enrichment Ratio	FDR
BNT162b2 Female Recipients	Downregulation of SMAD2/3:SMAD4 transcriptional activity	32.6610577	0.0475592
	Negative regulators of DDX58/IFIH1 signaling	24.6141304	0.0284525
	Synthesis, secretion, and deacylation of Ghrelin	35.3828125	0.0475592
	TICAM1, RIP1-mediated IKK complex recruitment	30.328125	0.04755928
	Downregulation of SMAD2/3:SMAD4 transcriptional activity	32.6610577	0.04755928
	Negative regulators of DDX58/IFIH1 signaling	24.6141304	0.0284525
	Synthesis, secretion, and deacylation of Ghrelin	35.3828125	0.0475592
6-month			
Cohort	Pathway	Enrichment Ratio	FDR
BNT162b2 Female Recipients	Eukaryotic Translation Elongation	8.5131579	0.01429319
	Eukaryotic Translation Termination	7.402746	0.04432762
	Glycolysis	6.00928793	0.04205984
	Infectious disease	3.11456996	0.01609754
	Influenza Infection	5.29707602	0.03293579
	Innate Immune System	1.8888979	0.01609754
	Metabolism of RNA	3.10982937	0.00260869
	mRNA Splicing	5.20248538	0.00260869
	mRNA Splicing - Major Pathway	5.35112782	0.00260869
	Nonsense Mediated Decay (NMD) enhanced by the Exon Junction Complex (EJC)	7.04537205	0.023371
	Nonsense Mediated Decay (NMD) independent of the Exon Junction Complex (EJC)	7.09429825	0.04744206
	Nonsense-Mediated Decay (NMD)	7.04537205	0.023371
	Peptide chain elongation	9.72932331	0.00743626
	Processing of Capped Intron-Containing Pre-mRNA	4.98331194	0.00260869
	Purine metabolism	4.00619195	0.023371
	Purine ribonucleoside monophosphate biosynthesis	17.0263158	0.04432762
	Regulation of expression of SLITs and ROBOs	4.19109312	0.04744206
	Selenocysteine synthesis	8.10776942	0.03293579
	Signaling by ROBO receptors	3.90186404	0.01854041
	Viral mRNA Translation	8.5131579	0.02911311
1-month			
Cohort	Pathway	Enrichment Ratio	FDR
mRNA-1273 Male Recipients	None detected	NA	NA
6-month			
Cohort	Pathway	Enrichment Ratio	FDR
mRNA-1273 Male Recipients	Activation of the mRNA upon binding of the cap-binding complex and eIFs, and subsequent binding to 43S	7.41395539	6.16E-04
	Cap-dependent Translation Initiation	9.03575813	3.84E-07
	Eukaryotic Translation Elongation	8.34069982	4.07E-04
	Eukaryotic Translation Initiation	9.03575813	3.84E-07
	Eukaryotic Translation Termination	7.61542157	0.00141979
	Formation of a pool of free 40S subunits	8.00707182	4.43E-04
	Formation of the ternary complex, and subsequently, the 43S complex	7.50662983	0.00521615
	GTP hydrolysis and joining of the 60S ribosomal subunit	8.83132922	1.26E-06
	HIV Infection	2.94377641	0.02549153
	HIV Life Cycle	4.09452536	0.01282603
	Infectious disease	3.3566231	1.09E-04
	Influenza Infection	5.56046654	6.58E-04
	Influenza Life Cycle	5.77433064	0.00114598
	Influenza Viral RNA Transcription and Replication	5.83848987	0.00704071
	Interconversion of nucleotide di- and triphosphates	6.82420894	0.00819223
	Intracellular signaling by second messengers	2.53199816	0.01448855
	ISG15 antiviral mechanism	4.69164365	0.04808094
	L13a-mediated translational silencing of Ceruloplasmin expression	9.09894525	1.13E-06
	M Phase	2.72968358	0.04657549
	Major pathway of rRNA processing in the nucleolus and cytosol	5.41018366	0.00471349
	Metabolism	3.19917253	8.21E-06
	Metabolism of nucleotides	4.58151117	4.43E-04
	Metabolism of porphyrins	10.0088398	0.01798475
	Metabolism of RNA	3.19917253	8.21E-06
	mRNA Splicing	4.51787907	4.43E-04
	mRNA Splicing - Major Pathway	4.64696133	4.34E-04
	Nonsense Mediated Decay (NMD) enhanced by the Exon Junction Complex (EJC)	6.90264812	8.70E-04
	Nonsense Mediated Decay (NMD) independent of the Exon Junction Complex (EJC)	7.29811234	0.00179384
	Nonsense-Mediated Decay (NMD)	6.90264812	8.70E-04
	Peptide chain elongation	9.53222836	1.56E-04
	PI5P Regulates TP53 Acetylation	15.0132597	0.02311676
	PIP3 activates AKT signaling	2.43721748	0.04133459
	Processing of Capped Intron-Containing Pre-mRNA	4.57721331	1.39E-04
	Protein ubiquitination	5.26781041	0.00539683
	Purine metabolism	3.23815405	0.01974497
	Purine ribonucleoside monophosphate biosynthesis	12.5110497	0.04201052
	Regulation of expression of SLITs and ROBOs	4.23450914	0.00255575
	Regulation of TP53 Activity through Acetylation	15.0132597	0.02311676
	Ribosomal scanning and start codon recognition	7.41395539	6.16E-04
	Ribosome	5.65015149	0.00819223
	RNA transport	5.08261395	1.81E-04
	rRNA processing	4.44837324	0.01450373
	rRNA processing in the nucleus and cytosol	4.88236087	0.00819223
	Selenoamino acid metabolism	5.15160871	0.01410805
	Selenocysteine synthesis	8.34069982	8.58E-04
	Signaling by ROBO receptors	3.64905617	0.00142896
	Spliceosome	3.6395781	0.04657549
	SRP-dependent cotranslational protein targeting to membrane	5.83848987	0.00704071
	Synthesis of active ubiquitin: roles of E1 and E2 enzymes	5.77433064	0.01811131
	Translation	4.22059509	4.43E-04
	Translation initiation complex formation	7.69910752	5.05E-04
	Viral mRNA Translation	8.75773481	6.54E-04
1-month			
Cohort	Pathway	Enrichment Ratio	FDR
BNT162b2 Male Recipients	APC/C:Cdc20 mediated degradation of Cyclin B	100.644444	0.03847957
	APC-Cdc20 mediated degradation of Nek2A	111.827161	0.03595134
	Biological oxidations	19.3547009	0.01611921
	Chemical carcinogenesis	35.1085271	0.02213348
	Downregulation of SMAD2/3:SMAD4 transcriptional activity	77.4188034	0.04931422
	Drug metabolism	45.7474748	0.01611921
	Ethanol oxidation	91.4949495	0.04110933
	Phase I - Functionalization of compounds	41.9351852	0.01611921
	RA biosynthesis pathway	71.8888889	0.04931422
	TICAM1, RIP1-mediated IKK complex recruitment	71.8888889	0.04931422
6-month			
BNT162b2 Male Recipients	E3 ubiquitin ligases ubiquitinate target proteins	12.5024155	0.04476852
	Eukaryotic Translation Elongation	10.9396135	0.04476852
	Eukaryotic Translation Termination	11.4152489	0.04476852
	Formation of a pool of free 40S subunits	10.502029	0.04958801
	Influenza Infection	8.75169082	0.03633001
	Influenza Life Cycle	8.41508733	0.04476852
	Metabolism	1.88935756	0.04476852
	Metabolism of RNA	3.59658527	0.03959885
	Negative regulators of DDX58/IFIH1 signaling	11.4152489	0.04476852
	Nonsense Mediated Decay (NMD) enhanced by the Exon Junction Complex (EJC)	11.3168416	0.03633001
	Nonsense Mediated Decay (NMD) independent of the Exon Junction Complex (EJC)	10.9396135	0.04476852
	Nonsense-Mediated Decay (NMD)	11.3168416	0.03633001
	Peptide chain elongation	12.5024155	0.04476852
	Processing of Capped Intron-Containing Pre-mRNA	5.60321668	0.04476852
	Regulation of expression of SLITs and ROBOs	6.05886288	0.04476852
	Selenocysteine synthesis	12.5024155	0.04476852
	Viral mRNA Translation	13.1275362	0.04476852
B. Downregulated Pathways			
1-month					
Cohort	Pathway	Enrichment Ratio	FDR
mRNA-1273 Female Recipients	Neurotrophin signaling pathway	2.19549862	0.04949487
	Adaptive Immune System	1.47596546	0.04947068
	FoxO signaling pathway	2.13847268	0.04814867
	SHC1 events in ERBB2 signaling	3.76371191	0.04814867
	L13a-mediated translational silencing of Ceruloplasmin expression	3.04138336	0.04814867
	Retrograde neurotrophin signaling	5.5758695	0.04814867
	EGFR Transactivation by Gastrin	5.5758695	0.04814867
	SHC-related events triggered by IGF1R	5.5758695	0.04814867
	Unblocking of NMDA receptors, glutamate binding and activation	5.5758695	0.04814867
	CREB phosphorylation through the activation of CaMKII	5.5758695	0.04814867
	Ras activation upon Ca2+ influx through NMDA receptor	5.5758695	0.04814867
	CD209 (DC-SIGN) signaling	4.48060942	0.04814867
	RHO GTPases activate PAKs	5.5758695	0.04814867
	Activated NTRK2 signals through RAS	5.5758695	0.04814867
	Choline metabolism in cancer	2.66929923	0.04755468
	Ion homeostasis	3.96180201	0.04009573
	mTOR signaling pathway	2.28103752	0.03906048
	Olfactory transduction	4.82527168	0.03519761
	Neurotransmitter receptors and postsynaptic signal transmission	2.78793475	0.03519761
	Listeria monocytogenes entry into host cells	4.82527168	0.03519761
	Signaling by NTRK3 (TRKC)	4.82527168	0.03519761
	B cell receptor signaling pathway	2.65389943	0.03355829
	MET activates RAS signaling	6.27285319	0.0335385
	MET activates RAP1 and RAC1	6.27285319	0.0335385
	Activated NTRK3 signals through RAS	6.27285319	0.0335385
	Signaling to ERKs	3.65916436	0.03257863
	Translation	2.26729633	0.03238849
	Epithelial cell signaling in Helicobacter pylori infection	3.05165831	0.03211109
	Oncogenic MAPK signaling	3.05165831	0.03211109
	Proteoglycans in cancer	1.96611816	0.03114849
	Signaling by ERBB2	2.91760613	0.0271399
	Eukaryotic Translation Initiation	3.13642659	0.02712832
	Cap-dependent Translation Initiation	3.13642659	0.02712832
	Synaptic vesicle cycle	3.81825846	0.02674081
	Fc gamma R-mediated phagocytosis	2.64120134	0.02553008
	Long-term potentiation	3.46088452	0.02553008
	FCERI mediated Ca+2 mobilization	4.42789637	0.02553008
	Signaling by RAS mutants	3.46088452	0.02553008
	Regulation of signaling by CBL	4.42789637	0.02553008
	Cholinergic synapse	3.22603878	0.02372978
	ErbB signaling pathway	2.47380126	0.02190311
	GRB2 events in EGFR signaling	7.16897507	0.02162445
	Cell-extracellular matrix interactions	7.16897507	0.02162445
	MAP2K and MAPK activation	3.58448754	0.02162445
	Response to elevated platelet cytosolic Ca2+	2.19245354	0.02153177
	Adherens junction	2.87505771	0.02148228
	Glioma	3.05992838	0.02148228
	Signaling by BRAF and RAF fusions	3.32092228	0.02071388
	GTP hydrolysis and joining of the 60S ribosomal subunit	3.32092228	0.02071388
	Tie2 Signaling	4.70463989	0.02048885
	Role of LAT2/NTAL/LAB on calcium mobilization	5.70259381	0.02025908
	Chemokine signaling pathway	2.16698565	0.01985779
	TGF-beta receptor signaling activates SMADs	4.18190212	0.01863291
	Signaling by moderate kinase activity BRAF mutants	3.71724633	0.01863291
	Paradoxical activation of RAF signaling by kinase inactive BRAF	3.71724633	0.01863291
	Activation of the mRNA upon binding of the cap-binding complex and eIFs, and subsequent binding to 43S	3.71724633	0.01863291
	Regulation of actin dynamics for phagocytic cup formation	3.42155628	0.01829587
	Golgi-to-ER retrograde transport	3.00006022	0.01693218
	Signaling by NTRK1 (TRKA)	2.84053729	0.016891
	EPH-Ephrin signaling	2.71823638	0.01625992
	Downstream signal transduction	3.86021735	0.01563779
	Translation initiation complex formation	3.86021735	0.01563779
	Platelet Aggregation (Plug Formation)	3.86021735	0.01563779
	Formation of Incision Complex in GG-NER	4.39099723	0.01495995
	Signaling by Erythropoietin	4.39099723	0.01495995
	MET receptor recycling	6.27285319	0.01446396
	Signaling by VEGF	2.66121044	0.01417322
	Shigellosis	3.13642659	0.0129849
	DAP12 interactions	3.64230185	0.0129849
	Signaling by high-kinase activity BRAF mutants	4.01462604	0.0129849
	Regulation of actin cytoskeleton	2.19549862	0.01177427
	Pathogenic Escherichia coli infection	3.76371191	0.01048107
	Fc epsilon RI signaling pathway	3.07241789	0.00964524
	Innate Immune System	1.44981198	0.00936362
	GAB1 signalosome	6.96983687	0.00893579
	Deadenylation of mRNA	6.96983687	0.00893579
	Macroautophagy	3.89349508	0.00870537
	RHO GTPases Activate WASPs and WAVEs	4.87888581	0.00870537
	Regulation of KIT signaling	5.79032602	0.00829642
	FCERI mediated MAPK activation	4.36372396	0.00827695
	Neutrophil degranulation	1.7219597	0.00827695
	Fcgamma receptor (FCGR) dependent phagocytosis	3.20315907	0.00743717
	Endosomal Sorting Complex Required For Transport (ESCRT)	5.16587909	0.00639158
	Signaling by NTRK2 (TRKB)	4.56207504	0.00624723
	Interferon Signaling	2.53733387	0.00615989
	PECAM1 interactions	6.27285319	0.00552616
	Ribosomal scanning and start codon recognition	4.18190212	0.00552616
	SHC1 events in EGFR signaling	7.84106648	0.00547587
	Focal adhesion	2.2303478	0.00504074
	Budding and maturation of HIV virion	5.48874654	0.00504074
	Signaling by NTRKs	2.89516301	0.00504074
	VEGFA-VEGFR2 Pathway	3.02827395	0.00504074
	Interleukin-3, Interleukin-5 and GM-CSF signaling	3.63165184	0.00504074
	Antigen activates B Cell Receptor (BCR) leading to generation of second messengers	4.77931671	0.00504074
	Oxytocin signaling pathway	3.08140157	0.00474565
	Bacterial invasion of epithelial cells	3.50112736	0.00403216
	RET signaling	4.51645429	0.00363104
	EGFR downregulation	5.85466297	0.00347741
	Trafficking of AMPA receptors	5.85466297	0.00347741
	Glutamate binding, activation of AMPA receptors and synaptic plasticity	5.85466297	0.00347741
	Integrin alphaIIb beta3 signaling	5.28240268	0.00279959
	Integrin signaling	5.28240268	0.00279959
	Vasopressin-regulated water reabsorption	4.90918945	0.00201276
	Platelet activation	3.24457923	0.00183056
	Signaling by Receptor Tyrosine Kinases	1.87314366	0.00176539
	RHO GTPase Effectors	2.57949103	0.00159805
	Translocation of SLC2A4 (GLUT4) to the plasma membrane	4.64655792	0.00148823
	Costimulation by the CD28 family	3.96180201	0.00148823
	ISG15 antiviral mechanism	4.31258657	0.00141757
	Insulin signaling pathway	3.01096953	0.00115283
	DAP12 signaling	5.3767313	0.00115283
	CD28 co-stimulation	5.3767313	0.00115283
	Signaling by MET	3.42155628	0.00115283
	Cargo recognition for clathrin-mediated endocytosis	3.42155628	0.00115283
	Erythropoietin activates RAS	7.31832872	0.00102824
	Antiviral mechanism by IFN-stimulated genes	4.30138504	8.58E-04
	Hemostasis	1.84922262	7.49E-04
	Tight junction	3.58448754	4.71E-04
	Signaling by Rho GTPases	2.49195538	3.24E-04
	GPVI-mediated activation cascade	5.5201108	1.51E-04
	Signaling by EGFR	5.26110267	3.34E-05
	Signaling by SCF-KIT	5.01828255	2.52E-05
	Clathrin-mediated endocytosis	3.58448754	1.07E-05
	RNA transport	3.92053324	9.13E-06
	Platelet activation, signaling and aggregation	2.80525111	1.25E-06
	TBC/RABGAPs	6.75538035	3.21E-07
	RAB GEFs exchange GTP for GDP on RABs	6.10331661	1.39E-08
	Rab-regulation of trafficking	5.80819739	1.05E-11
	Endocytosis	3.81825846	1.48E-12
	Membrane Trafficking	3.30150168	0
	Vesicle-mediated transport	2.98907769	0
	RAB geranylgeranylation	7.84106648	0
6-month			
Cohort	Pathway	Enrichment Ratio	FDR
mRNA-1273 Female Recipients	FCERI mediated Ca+2 mobilization	6.8662826	0.03823944
	GPVI-mediated activation cascade	5.6028866	0.03663942
	RET signaling	5.6028866	0.03663942
	Budding and maturation of HIV virion	7.29542526	0.03176858
	Signaling by NTRKs	3.59159397	0.03081647
	Retrograde neurotrophin signaling	10.3757159	0.02984514
	trans-Golgi Network Vesicle Budding	4.4467354	0.02984514
	Clathrin derived vesicle budding	4.4467354	0.02984514
	EGFR downregulation	7.78178694	0.0292749
	DAP12 signaling	6.67010309	0.01943979
	Golgi-to-ER retrograde transport	4.5675706	0.01283571
	Signaling by SCF-KIT	5.33608247	0.01045592
	Endosomal Sorting Complex Required For Transport (ESCRT)	8.23953912	0.00630182
	Signaling by EGFR	6.02460925	0.00474016
	Platelet activation, signaling and aggregation	2.90004482	0.00279803
	Cargo recognition for clathrin-mediated endocytosis	4.66907217	0.00279803
	Clathrin-mediated endocytosis	3.94142456	0.00279803
	Antigen activates B Cell Receptor (BCR) leading to generation of second messengers	7.78178694	0.00279803
	RAB GEFs exchange GTP for GDP on RABs	6.94051268	5.31E-05
	TBC/RABGAPs	8.97898493	1.38E-05
	RAB geranylgeranylation	7.58724227	1.64E-06
	Endocytosis	4.39840132	2.97E-08
	Rab-regulation of trafficking	7.78178694	1.54E-09
	Vesicle-mediated transport	3.6036463	2.29E-12
	Membrane Trafficking	4.03716766	0
1-month			
Cohort	Pathway	Enrichment Ratio	FDR
BNT162b2 Female Recipients	Signaling by NTRK2 (TRKB)	4.39566483	0.04439593
	Constitutive Signaling by EGFRvIII	5.37247924	0.0414074
	Signaling by EGFRvIII in Cancer	5.37247924	0.0414074
	Response to elevated platelet cytosolic Ca2+	2.34719967	0.04084622
	Retrograde neurotrophin signaling	7.16330565	0.03888623
	GAB1 signalosome	7.16330565	0.03888623
	Signaling by NTRK1 (TRKA)	3.04102599	0.03888623
	SHC-related events triggered by IGF1R	7.16330565	0.03888623
	CD28 co-stimulation	4.60498221	0.03888623
	Deadenylation of mRNA	7.16330565	0.03888623
	COPI-mediated anterograde transport	3.58165283	0.03888623
	Activated NTRK2 signals through RAS	7.16330565	0.03888623
	Signaling by the B Cell Receptor (BCR)	2.68623962	0.03888623
	Intra-Golgi and retrograde Golgi-to-ER traffic	2.76298932	0.0338327
	Formation of Incision Complex in GG-NER	4.83523132	0.03239498
	Ribosomal scanning and start codon recognition	4.17859497	0.03239498
	trans-Golgi Network Vesicle Budding	3.45373666	0.02926044
	Clathrin derived vesicle budding	3.45373666	0.02926044
	MET activates RAS signaling	8.05871886	0.02919414
	Activated NTRK3 signals through RAS	8.05871886	0.02919414
	Signaling by Rho GTPases	2.20786818	0.02151158
	Signaling by NTRKs	2.97552696	0.01987525
	Immune System	1.340816	0.01754536
	GRB2 events in EGFR signaling	9.20996441	0.01745487
	DAP12 interactions	4.15933877	0.01745487
	Cell-extracellular matrix interactions	9.20996441	0.01745487
	Signaling by MET	3.22348754	0.01745487
	MHC class II antigen presentation	3.50379081	0.01711049
	FCERI mediated Ca+2 mobilization	5.68850743	0.01711049
	Costimulation by the CD28 family	3.81728788	0.01711049
	Interleukin-3, Interleukin-5 and GM-CSF signaling	3.81728788	0.01711049
	Synaptic vesicle cycle	4.90530713	0.01622817
	Vasopressin-regulated water reabsorption	4.90530713	0.01622817
	FCERI mediated MAPK activation	4.90530713	0.01622817
	Interferon Signaling	2.71642209	0.01622817
	Role of LAT2/NTAL/LAB on calcium mobilization	7.32610806	0.01615184
	Tie2 Signaling	6.04403915	0.01413792
	Signaling by Receptor Tyrosine Kinases	1.90275306	0.00903642
	Hemostasis	1.81670541	0.00821089
	Signaling by Erythropoietin	5.6411032	0.00775507
	ISG15 antiviral mechanism	4.53302936	0.0060236
	GPVI-mediated activation cascade	5.15758007	0.00561733
	Golgi-to-ER retrograde transport	3.85416989	0.00560701
	Adaptive Immune System	1.81372701	0.00364644
	Neutrophil degranulation	1.94884051	0.00350327
	SHC1 events in EGFR signaling	10.0733986	0.00332732
	Endosomal Sorting Complex Required For Transport (ESCRT)	6.636592	0.00305047
	Antiviral mechanism by IFN-stimulated genes	4.60498221	0.00283646
	RNA transport	3.5256895	0.00234402
	Budding and maturation of HIV virion	7.051379	0.00220985
	DAP12 signaling	6.13997628	0.00201177
	Antigen activates B Cell Receptor (BCR) leading to generation of second messengers	6.13997628	0.00201177
	Innate Immune System	1.63906146	0.00175534
	EGFR downregulation	7.52147094	0.00168712
	Signaling by SCF-KIT	5.06548043	6.24E-04
	Erythropoietin activates RAS	9.40183867	2.85E-04
	Cargo recognition for clathrin-mediated endocytosis	4.39566483	1.04E-04
	Platelet activation, signaling and aggregation	2.80303265	6.84E-05
	RAB GEFs exchange GTP for GDP on RABs	5.66288352	2.71E-05
	Signaling by EGFR	6.23900815	2.70E-05
	Clathrin-mediated endocytosis	4.18634746	5.29E-06
	TBC/RABGAPs	7.43881741	3.27E-06
	RAB geranylgeranylation	6.44697509	1.77E-07
	Rab-regulation of trafficking	5.96942138	7.47E-09
	Vesicle-mediated transport	3.02878024	3.82E-12
	Endocytosis	4.43813503	5.73E-13
	Membrane Trafficking	3.39314478	0
6-month			
Cohort	Pathway	Enrichment Ratio	FDR
BNT162b2 Female Recipients	ER to Golgi Anterograde Transport	4.01586207	0.04233504
	COPI-independent Golgi-to-ER retrograde traffic	7.61176471	0.04233504
	Intra-Golgi and retrograde Golgi-to-ER traffic	3.69714286	0.04233504
	Endosomal Sorting Complex Required For Transport (ESCRT)	7.61176471	0.04233504
	Cargo recognition for clathrin-mediated endocytosis	4.23490909	0.03679775
	Clathrin-mediated endocytosis	3.69714286	0.02893682
	Platelet activation, signaling and aggregation	2.73267081	0.02789041
	RAB geranylgeranylation	5.823	0.0037292
	Golgi-to-ER retrograde transport	5.62608696	0.00200224
	TBC/RABGAPs	8.95846154	9.53E-05
	Rab-regulation of trafficking	6.23037037	3.27E-05
	Endocytosis	4.1257971	4.77E-06
	Vesicle-mediated transport	3.38697987	3.19E-09
	Membrane Trafficking	3.79443609	1.50E-10
1-month			
Cohort	Pathway	Enrichment Ratio	FDR
mRNA-1273 Male Recipients	None Detected	ND	ND
6-month			
Cohort	Pathway	Enrichment Ratio	FDR
mRNA-1273 Male Recipients	Peptide ligand-binding receptors	10.228094	0.04627729
	Post-translational protein phosphorylation	10.1048639	0.04627729
	Complement and coagulation cascades	12.333878	0.0353268
	Activation of C3 and C5	71.8888889	0.01119387
1-month			
Cohort	Pathway	Enrichment Ratio	FDR
BNT162b2 Male Recipients	DAP12 signaling	6.38067061	0.04961712
	Signaling by MET	3.89800968	0.04961712
	Erythropoietin activates RAS	8.93293886	0.04496226
	SUMO is transferred from E1 to E2 (UBE2I, UBC9)	16.0792899	0.03298049
	Integrin alphaIIb beta3 signaling	7.05232015	0.03298049
	Integrin signaling	7.05232015	0.03298049
	RHO GTPases Activate WASPs and WAVEs	7.44411571	0.02818226
	Response to elevated platelet cytosolic Ca2+	3.12219222	0.02818226
	Signaling by Receptor Tyrosine Kinases	2.14018327	0.02818226
	InlB-mediated entry of Listeria monocytogenes into host cell	10.7195266	0.02697305
	FCERI mediated Ca+2 mobilization	7.88200487	0.02517852
	Golgi-to-ER retrograde transport	4.66066375	0.02235289
	Negative regulation of MET activity	8.37463018	0.02035789
	RNA transport	4.18731509	0.0107186
	Antigen activates B Cell Receptor (BCR) leading to generation of second messengers	7.65680473	0.00903136
	Formation of Incision Complex in GG-NER	8.03964497	0.00709878
	RAB GEFs exchange GTP for GDP on RABs	5.79433872	0.00598092
	Cargo recognition for clathrin-mediated endocytosis	4.8725121	0.00362047
	Clathrin-mediated endocytosis	4.17643895	0.00313745
	Endosomal Sorting Complex Required For Transport (ESCRT)	9.45840585	0.00313745
	Budding and maturation of HIV virion	10.0495562	0.00249234
	Signaling by EGFR	6.91582363	0.0023029
	EGFR downregulation	10.7195266	0.00200892
	Platelet activation, signaling and aggregation	3.16259326	0.00152845
	RAB geranylgeranylation	6.69970414	3.70E-04
	TBC/RABGAPs	9.27651343	7.06E-05
	Rab-regulation of trafficking	6.94784133	2.25E-06
	Vesicle-mediated transport	3.41729876	3.09E-09
	Endocytosis	5.24324672	3.23E-10
	Membrane Trafficking	3.82840237	2.38E-10
6-month			
Cohort	Pathway	Enrichment Ratio	FDR
BNT162b2 Male Recipients	Costimulation by the CD28 family	4.90758514	0.03955952
	RAB GEFs exchange GTP for GDP on RABs	5.04022258	0.03516174
	InlB-mediated entry of Listeria monocytogenes into host cell	10.6564706	0.03408549
	Signaling by SCF-KIT	5.32823529	0.03386844
	FCERI mediated Ca+2 mobilization	7.83564014	0.03386844
	Negative regulation of MET activity	8.32536765	0.03386844
	TBC/RABGAPs	6.1479638	0.03386844
	Golgi-to-ER retrograde transport	4.63324808	0.03386844
	Endosomal Sorting Complex Required For Transport (ESCRT)	7.83564014	0.03386844
	Signaling by MET	4.35946524	0.02951796
	Signaling by Receptor Tyrosine Kinases	2.22009804	0.02905208
	EGFR downregulation	10.6564706	0.00207877
	Cargo recognition for clathrin-mediated endocytosis	5.32823529	0.00105399
	Clathrin-mediated endocytosis	4.49786096	0.00105399
	Rab-regulation of trafficking	5.42690632	0.00105399
	Signaling by EGFR	7.7345351	5.09E-04
	Endocytosis	4.05409207	1.49E-05
	Vesicle-mediated transport	3.30779708	2.55E-08
	Membrane Trafficking	3.70572755	1.52E-09

Enrichment ratio was calculated using the WEB-based GEne SeT AnaLysis Toolkit (WebGestalt) 2 (30).FDR, False Discovery Rate.

**Figure 5:**
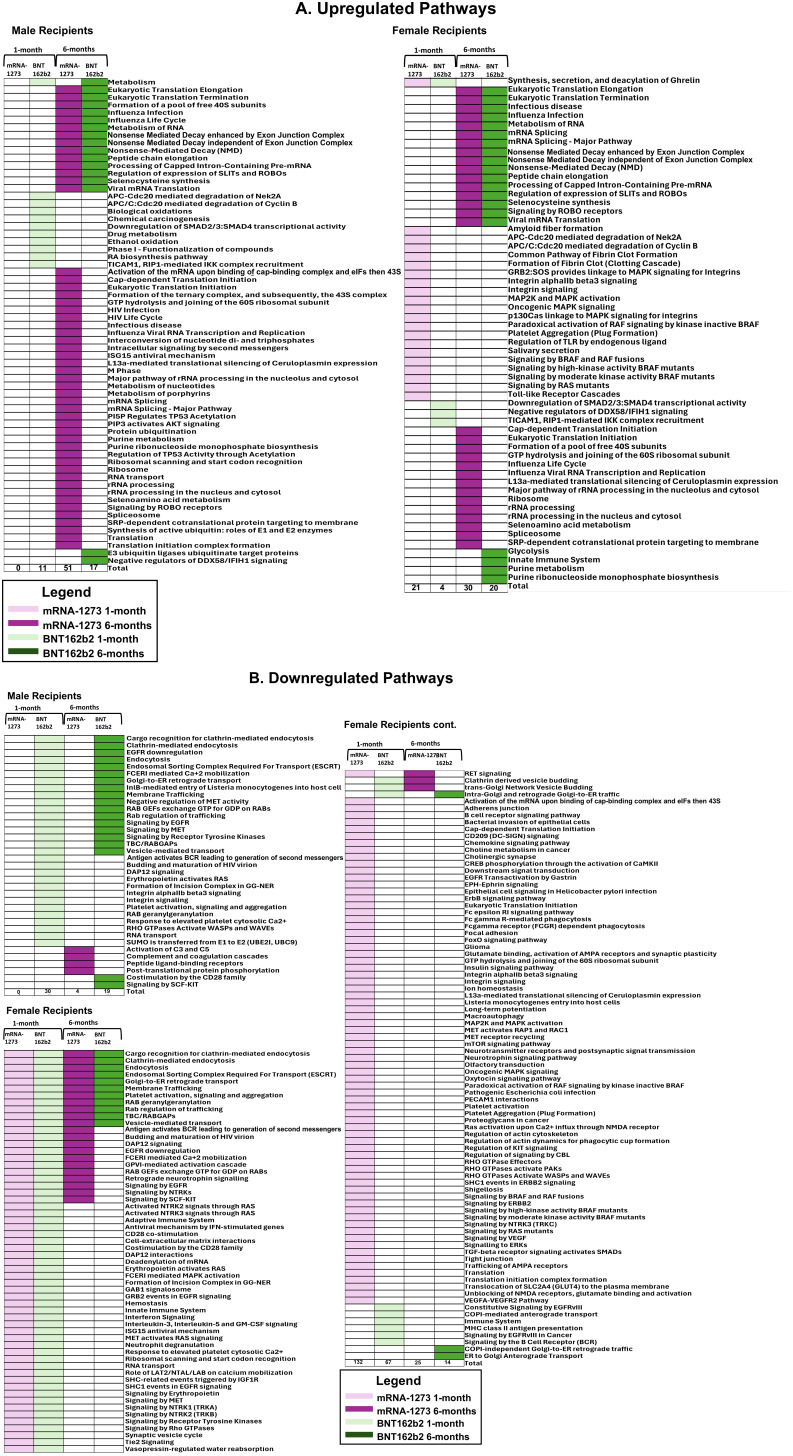
Cellular Pathways and Processes Impacted by Third Vaccination. Assessment of pathways modulated by vaccination. Upregulated pathways and cellular processes are presented in panel **(A)**, downregulated pathways in panel **(B)** Pathway assessments utilized both KEGG and REACTOME databases and identified processes are listed to the left of the figures. mRNA-1273 vaccination responses are indicated by purple, BNT162b2 indicated by green. Lighter colors indicate 1-month, darker colors 6-months. Common pathways between 1-month to 6-months and between BNT162b2 to mRNA-1273 vaccine recipient sera are indicated by the boxes.

Sera from male recipients of the mRNA-1273 vaccine demonstrated no significantly upregulated or downregulated pathways at 1-month, while sera from female recipients demonstrated an upregulation of 21 (including TLR signaling cascades, fibrin clot formation, and platelet aggregation) and downregulation of 132 pathways, (including membrane and vesical trafficking, platelet activation, and RNA transport) (**Figure 5**; **Table 5**; **Supplementary Table S2**).

At 6-months, sera from male recipients of mRNA-1273 demonstrated upregulation of 51 pathways (including class I antigen processing, metabolism of RNA, eucaryotic translation and peptide chain elongation) and downregulation of 4 (including complement activation, coagulation cascades, and peptide ligand receptors). Sera from female recipients of mRNA-1273 demonstrated upregulation of 30 pathways at 6-months (including class I antigen processing, metabolism of RNA, and peptide chain elongation) and downregulation of 25 (including endocytosis, membrane and vesicle trafficking and platelet activation) (**Table 5**; **Figure 5**; **Supplementary Table S2**).

One month after booster, the sera from male recipients of BNT162b2 demonstrated upregulation of 11 pathways (including APC-Cdc20 degradation and TICAM1 and RIP1 signaling) and downregulation of 30 (including platelet activation, membrane trafficking, and RNA transport), while sera from the female recipients demonstrated upregulation of 4 pathways (including TICAM1 and RIP1 mediated signaling) and downregulation of 67 (including membrane and vesical trafficking, platelet activation, and RNA transport) (**Table 5**; **Figure 5**; **Supplementary Table S2**). Upregulated pathways in sera from both male and female recipients of BNT162b2 included TICAM1, RIP1 mediated IKK signaling (**Figure 5**; **Supplementary Table S2**).

By 6-months post-third vaccination, sera from the male recipients of BNT162b2 showed upregulation of 17 pathways or processes associated with protein synthesis (mRNA processing and translation), regulation of cellular function (SLIT – ROBO pathway), and suppression of RIG-1 signaling pathway (DDX58/IFIH1) and downregulation of 19 (including membrane trafficking, endocytosis, and EGFR, RTK, MET, CD28, SCF-KIT, and ESCRT signaling). The sera from the female recipients of BNT162b2 demonstrated upregulation of 20 pathways and processes (including innate immune system activation, class I antigen processing, glycolysis and neddylation) and downregulation of 14 (including membrane and vesical trafficking, endocytosis, and platelet activation) (**Table 5**; **Figure 5**; **Supplementary Table S2**).

**Figure 6:**
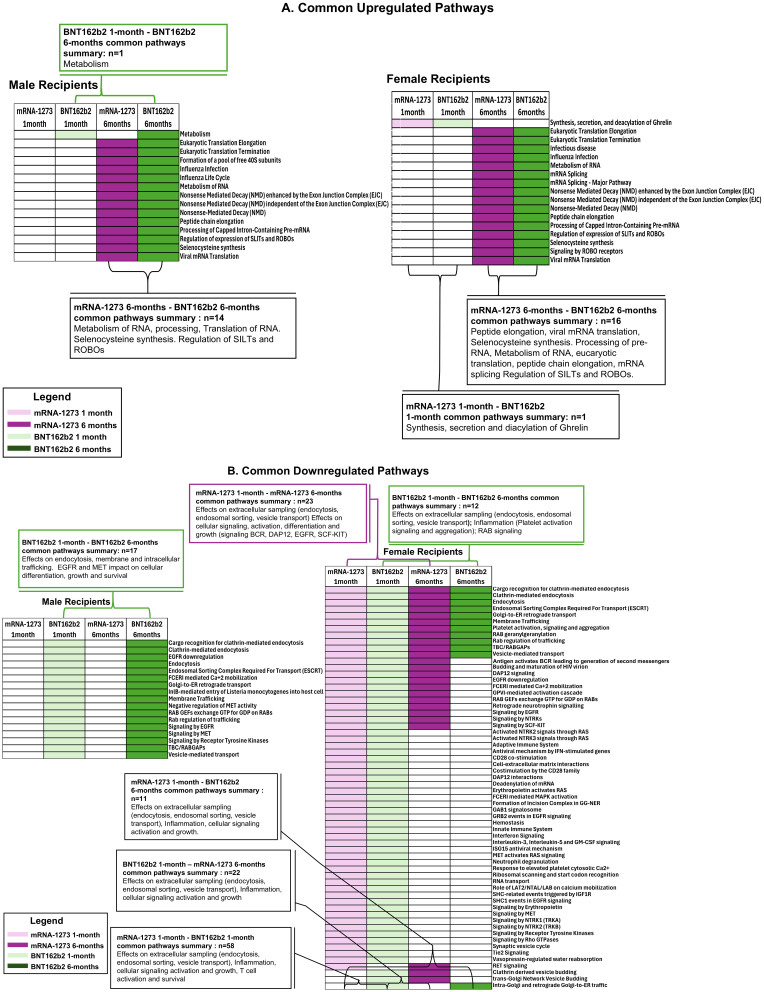
Common Pathways Modulated by Third Vaccination. Common cellular pathways and processes **(A)** upregulated or **(B)** downregulated according to BNT162b2 (green) and mRNA-1273 (purple) cohorts at 1-month (light green/purple) and 6-months (dark green/purple) after third vaccination. Number of common pathways (n) are indicated in the boxes.

We evaluated common trends detected in pathways impacted by vaccination and found 17 pathways, including membrane trafficking, endocytosis and EGFR signaling pathways, were downregulated in sera from male recipients of BNT162b2 at 1-month and at 6-months, and 1 upregulated (metabolism) (**Figure 6**; **Table 5**). Likewise, sera from female recipients of BNT162b2 demonstrated 12 common pathways downregulated at 1-month and 6-months, most notably platelet activation, vesicle trafficking, endocytosis and signaling by RAB (**Figure 6**; **Table 5**). No comparisons were possible between sera from the male recipients of BNT162b2 and mRNA-1273 at 1-month because no significant pathway changes were noted in the mRNA-1273 group even though there were significant marker changes (**Figure 6**; **Table 5**). There was 1 common pathway upregulated in sera from female recipients of either vaccine at 1-month post-third vaccination: synthesis, secretion, and deacylation of Ghrelin (**Figure 6**; **Table 5**). Sera from recipients of either vaccine, regardless of sex assigned at birth (except male recipients of mRNA-1273), demonstrated upregulation of translation, peptide chain elongation and RNA metabolism and processing at 6-months (**Figure 6**; **Table 5**).

### 3.4 Predictive modeling of serological responses

**Figure 7:**
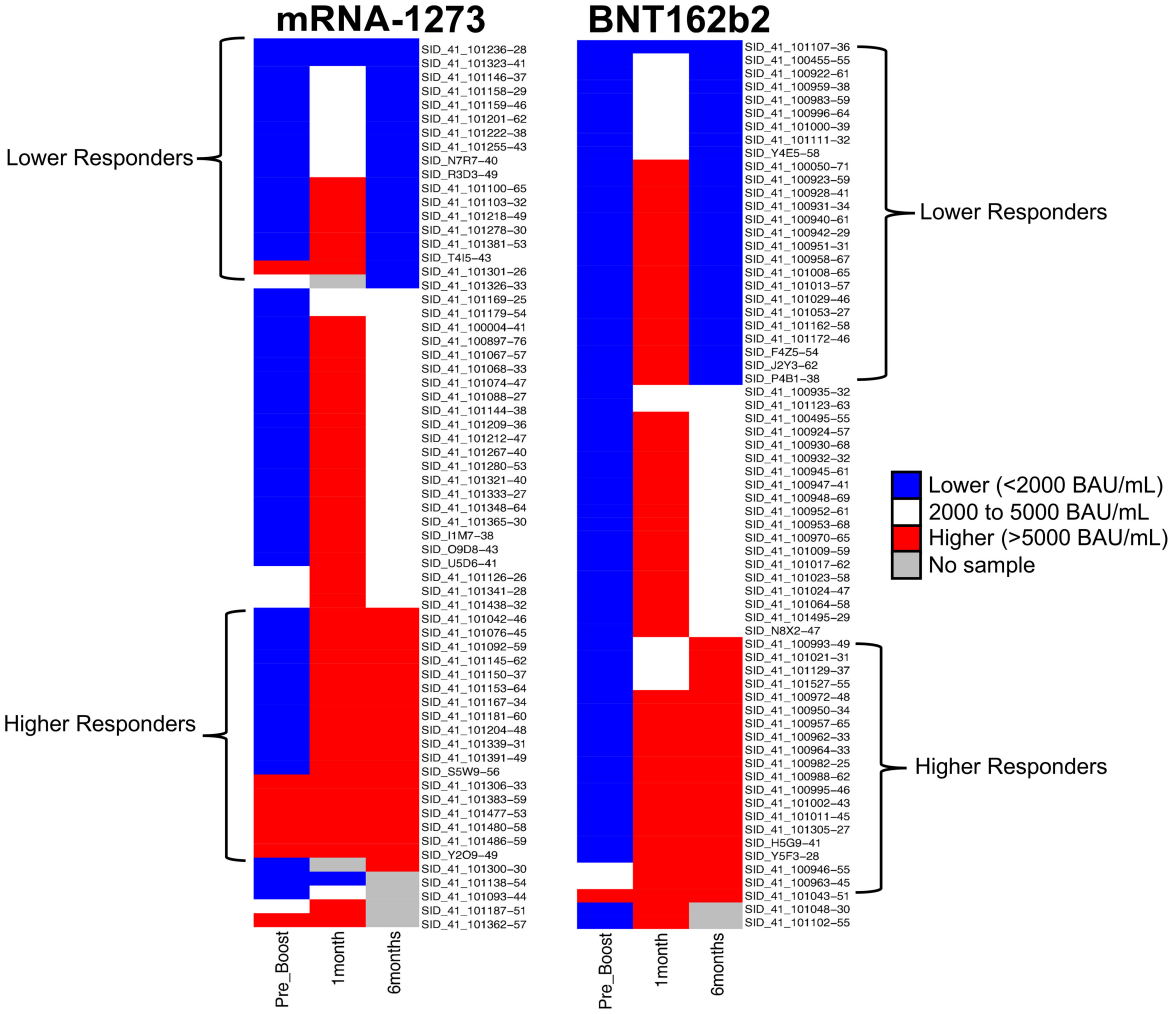
Serological Antibody Assessment of IgG to SARS-CoV-2 Spike S-2 Protein at Pre-Third Dose and 1, 6 Months after Homologous Third Vaccination. Serology Response (Antibody Content BAU/mL) Test Result Distribution. Test results are broken out into tertiles; Higher responses (red), lower responses (blue), middle responses (white). Grey indicates samples unavailable for testing and there are no data. Column 1: Pre-third vaccination; Column 2: 1-month; Column 3: 6-months. Column 4 lists vaccine-recipient identification numbers.

The overarching goal of this study was to investigate the possibility of identifying proteomic markers in pre-boost sera that are predictive of humoral response robustness to vaccination at later timepoints. Predictive modeling was performed as a pilot effort to investigate the utility of machine learning to identify markers or develop models of vaccine responsiveness (**Figure 1**). Change in anti-spike IgG antibody levels (BAU/mL) in pre-third vaccination and 6-month post-third vaccination sera were analyzed to identify vaccine-recipient samples with either “higher” or “lower” antibody content. The predictive potential of proteomic markers of avidity responses were not analyzed due to the limited range of AI measurements. The sera with the lowest and highest quartile of antibody titers were identified as “lower” and “higher” responders (**Figure 7**). Specifically, 44 vaccine-recipients with 6-month serum IgG anti-SARS-CoV-2 Spike antibody less than 2000 BAU/mL (7 male and 11 female recipients of mRNA-1273, 13 male and 13 female recipients of BNT162b2) were identified as relatively “Lower” responders, and 39 vaccine-recipients with sera containing greater than 5000 BAU/mL (9 male and 10 female recipients of mRNA-1273, and 8 male and 12 female recipients of BNT162b2) were identified as relatively “Higher” responders (**Figures 1**, **7**; **Table 6**). The demographics of these 2 populations were well-balanced concerning sex assigned at birth and vaccine-received (**Table 6**).

**Table 6:** Selected vaccine-recipient samples to develop model of 6-month post-third vaccination serology.

Lower Serology Responders (<2000 BAU/mL)			Higher Serology Responders (>5000 BAU/mL)		
Sex assigned at birth	Vaccine	Count	Sex assigned at birth	Vaccine	Count
Male	mRNA-1273	7	Male	mRNA-1273	9
Male	BNT162b2	13	Male	BNT162b2	8
Female	mRNA-1273	11	Female	mRNA-1273	10
Female	BNT162b2	13	Female	BNT162b2	12
**Total**		**44**	**Total**		**39**

Random Forest (RF) modeling, which combines outputs of multiple “decision trees” to reach a single result, was used for predictive modeling. Attempts to develop predictive models restricted by vaccine or sex assigned at birth did not return results with sufficient power due to the limitations of small cohort sizes. However, evaluation of the entire dataset as a single cohort returned a productive model that was statistically different from random selection (**Figure 1**).

An RF model with 85 marker values from the pre-third vaccination sera could predict higher (>5,000 BAU/ml) and lower (<2,000 BAU/mL) responders at month-6 with 79.17% accuracy (**Figure 1**). The associated markers and comparative changes are listed in **Supplementary Table S3** (**Figure 7**). Protein markers with the highest predictive power (i.e. markers that contributed the most to the model) were associated with complement cascade and activation, signaling by interleukins, tumor necrosis factor receptor (TNFR) apoptotic signaling, IL-17 signaling and phosphoinositide 3-kinase (PI3K) signaling (**Table 7**).

**Table 7:** Top 30 markers and pathways that contributed to the predictive model of 6-month post-third vaccination serology responses.

Sequence Identification	Protein Symbol	Gene Symbol	Name	Pathway
P25225.14	Q9BXU8	FTHL17	Ferritin heavy polypeptide-like 17	
23595.6	Q8IV20	LACC1	Laccase domain-containing protein 1	
7886.26	O15269	SPTLC1	Serine palmitoyltransferase 1	Sphingolipid *de novo* biosynthesis
7871.16	Q24JP5	TMEM132A	Transmembrane protein 132A	
3622.33	Q99538	LGMN	Legumain	Vitamin D (calciferol) metabolism
5837.49	P42702	LIFR	Leukemia inhibitory factor receptor	Signaling by Interleukins
2946.52	P00746	CFD	Complement factor D	Complement cascade, Alternative complement activation
21742.43	Q6GQQ9	OTUD7B	OTU domain-containing protein 7B	TNFR1-induced proapoptotic signaling
7857.22	P30990	NTS	Neurotensin/neuromedin N	
2312.13	HCE000483	HCE000483	HCE000483	
20087.3	O60613	SELENOF	15 kDa selenoprotein	
5708.1	Q969E1	LEAP2	Liver-expressed antimicrobial peptide 2	
14116.129	P26447	S100A4	Protein S100-A4	
23371.5	Q9GZT8	NIF3L1	NIF3-like protein 1	
3622.33	Q99538	LGMN	Legumain	Vitamin D (calciferol) metabolism
5837.49	P42702	LIFR	Leukemia inhibitory factor receptor	Signaling by Interleukins
20535.68	Q8NFR9	IL17RE	Interleukin-17 receptor E	IL-17 signaling pathway
11218.84	P51580	TPMT	Thiopurine S-methyltransferase	Metabolic disorders of biological oxidation enzymes, Methylation
9176.3	P15941	MUC1	Mucin-1: region 2	Termination of O-glycan biosynthesis
8039.41	Q8N128	FAM177A1	Protein FAM177A1	Signaling by Interleukins
7211.2	P07998	RNASE1	Ribonuclease pancreatic	
20120.101	Q86WK6	AMIGO1	Amphoterin-induced protein 1: Extracellular domain	
4133.54	P10144	GZMB	Granzyme B	Allograft rejection, Graft-vs-host
25236.11	Q96S19	METTL26	Methyltransferase-like 26	
7808.5	O94923	GLCE	D-glucuronyl C5-epimerase	
6232.54	P42081	CD86	T-lymphocyte activation antigen CD86	Allograft rejection, Graft-vs-host, CD28 dependent Vav1 pathway, CD28 dependent PI3K/Akt signaling
8091.16	P48740	MASP1	Mannan-binding lectin serine protease 1: Mannan-binding lectin serine protease 1 heavy chain	Lectin pathway of complement activation, Complement and coagulation cascades
3470.1	P16581	SELE	E-selectin	Cell adhesion molecules (CAMs)
17441.4	P09923	ALPI	Intestinal-type alkaline phosphatase	Thiamine metabolism
22969.12	P80098	CCL7	C-C motif chemokine 7	IL-17 signaling pathway

## 4 Discussion

The SARS-CoV-2 pandemic fueled the development and wide administration of 2 novel mRNA vaccines: mRNA-1273 and BNT162b2. These new vaccines have proven to be both effective and versatile, allowing for protection against severe disease caused by SARS-CoV-2 as well as being easily amendable to rapid adjustment for targeting new viral variants on a large scale (33, 34). Precipitous decreases in antibody levels after primary vaccination and lack of vaccine effectiveness against rapidly emerging variants affected the longevity of vaccine-imparted immunity, leading to booster recommendations for all BNT162b2 and mRNA-1273 recipients (7, 8, 11–13, 16, 17). However, studies are suggesting differences in COVID-19 vaccine efficacy in different populations (2–4, 6–10). Consequently, correlate(s) of protection or immunity are needed to help determining which and when populations need additional doses.

Traditionally, studies have looked to binding or neutralizing antibody levels as surrogate correlates of protection against various pathogens (20). However, correlates of protection against SARS-CoV-2 infection or severe disease are not yet fully established (35). In addition, studies have not yet comprehensively investigated if detection of systemic proteomic changes in the blood after vaccination can predict longitudinal immune responses.

The SARS-CoV-2 mRNA vaccines are known to activate both adaptive and innate immune responses due to their complex nature (36, 37). The lipid delivery systems of mRNA vaccines may have strong inflammatory effects (38, 39), as lipid nanoparticles can be detected by TLR-4 and TLR-2 (37, 40, 41). The RNA components could also trigger a variety of innate sentry sensors (TLR receptors) such as TLR-3, TLR-7, and TLR-8 (37, 42). Repetitive vaccination with mRNA vaccines may also have additional effects, as studies have demonstrated that repeated vaccinations can correlate with upregulation of dendritic cell activation and TLR signaling; BNT162b2 vaccination induces a moderate innate immune response that increases notably with subsequent vaccinations (36).

In this proof-of-concept study, we explored the feasibility of using proteomics to further analyze COVID-19 vaccine immunogenicity according to vaccine type, recipient sex assigned at birth, and time since third vaccination. Protein marker expression in pre-third vaccination sera were analyzed for predictiveness of robustness/weakness of antibody responses to vaccination by 6-months post-third vaccination.

The measured serologic responses to third vaccination doses were comparable irrespective of vaccine or sex assigned at birth of the recipient, however the proteomic assessments differed extensively. Proteomics marker modulation and pathway functional enrichment analyses revealed significant marker changes and process differences at the cohort level based on sex assigned at birth, including pathways related to RNA processing, protein synthesis, and cell cycle regulation. Interestingly, pathways associated with innate and adaptive immunity and inflammation were particularly evident in the proteomics data.

Our cohort level analyses identified 3 upregulated markers common to sera from recipients of mRNA-1273 vaccine regardless of sex assigned at birth: UB2D1/PolyUbiquitin K48 (UBE2D1|UBB), Insulin (INS) and Band 4.1-like protein1 (EPB41L1). Insulin is a hormone product of the INS gene (43). Secreted by pancreatic-beta cells, the primary role of insulin is to regulate energy levels by acting in the muscle and adipose tissues to mediate blood glucose deposition and storage (43, 44). The Band 4.1-like protein1 (EPB41L1) is an erythrocyte membrane protein, an important membrane skeletal protein that provides a connective bridge between the actin cytoskeleton and numerous trans-membrane proteins that function in cellular adhesion, migration and invasion (45, 46). Loss of EPB41L1 expression is reported in multiple cancer types and may play an important role in metastasis (45, 46). There are no available reports describing Band 4.1-like protein1 increase in serum following third vaccination of any type. However, there have been case reports of pancreatitis or development or worsening of diabetes following COVID-19 infection and vaccination with BNT162b2 or mRNA-1273, both of which could affect plasma insulin levels (47–53).

Sera from BNT162b2 vaccine cohorts, regardless of sex assigned at birth, showed upregulation of 3 common markers at 1-month post-third vaccination: UB2D1/PolyUbiquitin K48 (UBE2D1|UBB), Glutathione-specific gamma-glutamylcyclotransferase 1 (CHAC1), and Cancer/testis antigen 1 (CTAG1A|CTAG1B). Glutathione-specific gamma-glutamylcyclotransferase 1 is a proapoptotic endoplasmic reticulum (ER) stress protein (54). Overexpression of the enzyme results in glutathione depletion, which adversely impacts the regulation of the cellular oxidative balance between reactive oxygen species and antioxidant defenses (55). Cancer-testis antigen are antigens identified in a variety of malignant tumors and are normally only expressed in testis tissues (56). However, testis antigens have been identified in cancer tissues in both male and female patients and can be a primary target of anti-cancer immune responses (56). To date, there are no searchable reports describing modulation of either Glutathione-specific gamma-glutamylcyclotransferase 1 proteins or cancer-testis antigens in sera associated with either SARS-CoV-2 infection or vaccination with either BNT161b2 or mRNA-1273.

UB2D1/PolyUbiquitin K48 was increased in all vaccine cohorts at 1-month regardless of sex assigned at birth. The protein accumulates early in the oxidative stress response and binds oxidized proteins that are then targeted for removal through the ubiquitin/proteasome system (57). Additionally, k48-linked proteins can be detected following DNA damage, suggesting a role in protein degradation in that pathway as well (58). There are no searchable reports describing upregulation of UB2D1/PolyUbiquitin K48 protein in serum following either vaccination for or infection with SARS-CoV-2. However, oxidative stress has been associated with inflammation (59) and the induction of cytokine storm and tissue damage caused by SARS-CoV-2 infection (60). The current mRNA vaccines do have a recognized inflammatory component as described above.

In sera from female recipients of either vaccine at 1-month post-third vaccination, 7 markers (UBE2D1|UBB, CHAC1, LEP, CST5, CST2, INS) were upregulated and 342 downregulated. Leptin (LEP) is a hormone produced in adipose tissue and is involved in regulation of appetite, neuroendocrine function and energy homeostasis (61). Leptin has been shown to amplify inflammatory immune responses through the innate immune system by promoting cellular proliferation and survival, mediating secretion of mediators of inflammation, and migration of innate effector cells (62). While there are no reports of vaccine induced serum Leptin increases, there are reports of elevated plasma Leptin in intensive care patients with COVID-19 compared to healthy study participants (63). CST5 (or cystatin D) and CST2 (cystatin SA) are members of the cystatin superfamily of related proteins (64, 65). CST5, specifically, has been shown to be inhibitory against coronavirus replication, while CST2 acts as a protease inhibitor (64, 66) that protects against allergen, viral and bacterial proteases that can have a role in inflammatory tissue remodeling (67).

Sera from both male and female recipients of BNT162b2 demonstrated upregulated TICAM1, RIP1 mediated IKK signaling at 1-month. TICAM-1 is a molecule that has a role in TLR-3 signaling following double-stranded RNA detection. It physically binds the TIR domain of TLR-3 and activates the IFN-beta promoter I in response to dsRNA (68). Receptor-interacting serine/threonine-protein kinase-1 (RIP1) is a cellular kinase at the crossroads of inflammation signaling and cellular death, regulating pro-survival NF-KB signaling and inflammation, or, upon modification, promoting cellular death by binding death receptors signaling apoptosis or necrosis (69–73). IkB kinases (IKK signaling) are multiprotein complexes that regulate a diverse array of biological processes including innate immunity and inflammation (74).

Sera from female recipients of mRNA-1273 showed upregulation of multiple pathways 1 month after a third vaccination associated with innate immune activation, including Toll-like receptor (TLR) surveillance, and processes such as fibrin clot formation, amyloid fiber formation, and platelet aggregation (75–78). In addition, mitosis regulation (Nek2A degradation) was upregulated in sera from female recipients of mRNA-1273 and sera from male recipients of BNT162b2 at the 1-month timepoint. Receptor signaling through MAPK, MAP2K and RAS was also notedly enhanced in sera from the female recipients of mRNA-1273 at 1-month. These signals may indicate oxidative stress, as both the MAPK - MAP2K and RAS signaling pathways interact with reactive oxygen ions to play a role in promoting or suppressing tumorigenesis (79, 80). Specifically, the Raf-Ras-MEK1/2-ERK1/2 signaling pathway can promote tumorigenesis while the p38 mitogen activated protein kinases pathway (MAPK) suppresses cancer through oncogene-induced senescence, inflammation-induced senescence, contact inhibition, and DNA damage responses (79, 80). There were no searchable reports describing Nek2A degradation following vaccination for, or infection with SARS-CoV-2. MAPK signaling is enhanced in COVID-19 acute respiratory syndrome (81, 82), but there were no searchable reports that indicated enhancement following vaccination with BNT162b2 or mRNA-1273. While sera from male recipients of mRNA-1273 at 1-month demonstrated marker modulation that was statistically significant, those findings could not be mapped to statistically significant changes in somatic pathways or processes (**Figures 3**, **5**; **Supplementary Figures S1**, **S3**).

Proteomic assessments detected class I antigen processing and peptide chain elongation in sera from 6-months post-third vaccination in all cohorts irrespective of vaccine or sex assigned at birth (except in male recipients of BNT162b2), indicating antigen processing for CD8 T-cell activation (83). These observations are consistent with those of Zhang et al. (84), which demonstrated sustained T-cell responses 6 months after third vaccination with mRNA-1273 or BNT162b2 (84). In addition, sera from female recipients of BNT162b2 demonstrated innate immune system activation at 6-months. The activation of innate responses would be expected early after a third vaccination due to signaling through sentinel receptors like TLRs and RIG-I (85, 86), which would be unlikely at 6-months. It is important to note that as proteomic assessments are extremely sensitive and these results may have been reflective of normal immune surveillance encounters of “every-day” threats or pathogen associated molecular patterns (PAMP), and not specifically vaccine-associated events.

Our study results did not identify specific individual markers predictive of either robust or weaker IgG antibody responses at 6-months, but they did allow for the development of a predictive machine learning model. The small sample size limited the power of our results; assessments of protein marker abundances in pre-third vaccination sera to develop a working model were only productive when the entire dataset was evaluated irrespective of vaccine or sex assigned at birth. Assessments of pre-third vaccination serum protein markers identified 85 markers that predict SARS-CoV-2 spike IgG responses at 6-months with up to 79.17% accuracy (**Supplementary Table S3**). This data indicates that protein levels of these 85 markers in sera collected from individuals pre-third dose can be predictive of higher or lower immunological response at 6-months post-third-dose. Thirty of these markers were identified as top drivers of the predictive model, they were markers significantly associated with signaling by interleukins including, tumor necrosis factor receptor (TNFR), PI3 kinase, and IL-17. Additionally, there was a significant association with activation of the complement cascade. Complement cascade activation, TNFR pro-apoptotic, and IL-17 signaling are closely associated with inflammatory responses and may be the markers driving sub-optimal serology predictions by the model (87–89).

In this report, we investigate the utility of proteomic assessments of sera to evaluate or predict serologic vaccine responses. While our sample size is small and limits somewhat the analyses, we demonstrated that proteomic assessments have the potential to predict immunological response to third vaccination. Specifically, we were able to develop a model of vaccine responsiveness at 6-months even though we could not identify specific markers that were singularly predictive of the strength of the antibody response. While the serologic responses in male and female recipients of third vaccinations of mRNA vaccines were similar, the proteomic responses were clearly different, with sera from female recipients demonstrating higher responses compared to sera from male recipients. These differential responses were evident by the number, type, and abundance changes of protein markers and the associated molecular pathways and processes affected. This proof-of-concept study illustrates the utility of proteomics analyses in immunogenicity assessments to gain a better understanding of involved mechanisms. This study is small and observational, but also one of the first ones to assess proteomics changes observed following third vaccinations with either BNT162b2 or mRNA-1273 with the intent of modeling vaccine responsiveness. Further studies are needed to confirm the markers identified and reproduce these observations in larger populations to establish robust predictive models of immunity and protection.

## Data Availability

Original datasets are available in a publicly accessible repository: https://doi.org/10.6084/m9.figshare.c.7586102.

## References

[1] Rando HM Lordan R Kolla L Sell E Lee AJ Wellhausen N. The coming of age of nucleic acid vaccines during covid-19. mSystems. (2023) 8:e0092822. doi: 10.1128/msystems.00928-22 36861992 PMC10134841

[2] Crommelin DJA Anchordoquy TJ Volkin DB Jiskoot W Mastrobattista E. Addressing the cold reality of mrna vaccine stability. J Pharm Sci. (2021) 110:997–1001. doi: 10.1016/j.xphs.2020.12.006 33321139 PMC7834447

[3] Dickerman BA Gerlovin H Madenci AL Kurgansky KE Ferolito BR Figueroa Muniz MJ. Comparative effectiveness of bnt162b2 and mrna-1273 vaccines in U.S. Veterans. N Engl J Med. (2022) 386:105–15. doi: 10.1056/NEJMoa2115463 PMC8693691 34942066

[4] Naranbhai V Garcia-Beltran WF Chang CC Berrios Mairena C Thierauf JC Kirkpatrick G. Comparative immunogenicity and effectiveness of mrna-1273, bnt162b2, and ad26.Cov2.S covid-19 vaccines. J Infect Dis. (2022) 225:1141–50. doi: 10.1093/infdis/jiab593 PMC8689763 34888672

[5] Tenforde MW Self WH Adams K Gaglani M Ginde AA McNeal T. Association between mrna vaccination and covid-19 hospitalization and disease severity. Jama. (2021) 326:2043–54. doi: 10.1001/jama.2021.19499 PMC8569602 34734975

[6] Demonbreun AR Sancilio A Velez ME Ryan DT Pesce L Saber R. Covid-19 mrna vaccination generates greater immunoglobulin G levels in women compared to men. J Infect Dis. (2021) 224:793–7. doi: 10.1093/infdis/jiab314 PMC8536925 34117873

[7] Evans JP Zeng C Carlin C Lozanski G Saif LJ Oltz EM. Neutralizing antibody responses elicited by sars-cov-2 mrna vaccination wane over time and are boosted by breakthrough infection. Sci Transl Med. (2022) 14:eabn8057. doi: 10.1126/scitranslmed.abn8057 35166573 PMC8939766

[8] Hickey TE Kemp TJ Bullock J Bouk A Metz J Neish A. Sars-cov-2 igg spike antibody levels and avidity in natural infection or following vaccination with mrna-1273 or bnt162b2 vaccines. Hum Vaccin Immunother. (2023) 19:2215677. doi: 10.1080/21645515.2023.2215677 37264688 PMC10305493

[9] Pani A Cento V Vismara C Campisi D Di Ruscio F Romandini A. Results of the renaissance study: response to bnt162b2 covid-19 vaccine-short- and long-term immune response evaluation in health care workers. Mayo Clin Proc. (2021) 96:2966–79. doi: 10.1016/j.mayocp.2021.08.013 PMC8403667 34736776

[10] Richards NE Keshavarz B Workman LJ Nelson MR Platts-Mills TAE Wilson JM. Comparison of sars-cov-2 antibody response by age among recipients of the bnt162b2 vs the mrna-1273 vaccine. JAMA Netw Open. (2021) 4:e2124331. doi: 10.1001/jamanetworkopen.2021.24331 34473262 PMC8414189

[11] Doria-Rose N Suthar MS Makowski M O’Connell S McDermott AB Flach B. Antibody Persistence through 6 Months after the Second Dose of Mrna-1273 Vaccine for Covid-19. N Engl J Med. (2021) 384:2259–61. doi: 10.1056/NEJMc2103916 PMC8524784 33822494

[12] Naaber P Tserel L Kangro K Sepp E Jurjenson V Adamson A. Dynamics of antibody response to bnt162b2 vaccine after six months: A longitudinal prospective study. Lancet Reg Health Eur. (2021) 10:100208. doi: 10.1016/j.lanepe.2021.100208 34514454 PMC8418937

[13] Bullock JL Jr. Hickey TE Kemp TJ Metz J Loftus S Haynesworth K. Longitudinal assessment of bnt162b2- and mrna-1273-induced anti-sars-cov-2 spike igg levels and avidity following three doses of vaccination. Vaccines (Basel). (2024) 12:1-13. doi: 10.3390/vaccines12050516 PMC11125776 38793767

[14] Brito-Dellan N Tsoukalas N Font C. Thrombosis, cancer, and covid-19. Support Care Cancer. (2022) 30:8491–500. doi: 10.1007/s00520-022-07098-z PMC9106567 35567609

[15] Ku JH Sy LS Qian L Ackerson BK Luo Y Tubert JE. Vaccine effectiveness of the mrna-1273 3-dose primary series against covid-19 in an immunocompromised population: A prospective observational cohort study. Vaccine. (2023) 41:3636–46. doi: 10.1016/j.vaccine.2023.04.075 PMC10154542 37173268

[16] Atmar RL Lyke KE Deming ME Jackson LA Branche AR El Sahly HM. Homologous and heterologous covid-19 booster vaccinations. N Engl J Med. (2022) 386:1046–57. doi: 10.1056/NEJMoa2116414 PMC8820244 35081293

[17] FDA. Coronavirus (Covid-19) update: fda takes additional actions on the use of a booster dose for covid-19 vaccines: food and drug administration (2021). Available online at: https://www.fda.gov/news-events/press-announcements/coronavirus-covid-19-update-fda-takes-additional-actions-use-booster-dose-covid-19-vaccines (Accessed April 10, 2024).

[18] Gilbert PB Donis RO Koup RA Fong Y Plotkin SA Follmann D. A covid-19 milestone attained - a correlate of protection for vaccines. N Engl J Med. (2022) 387:2203–6. doi: 10.1056/NEJMp2211314 36507702

[19] Sadarangani M Marchant A Kollmann TR. Immunological mechanisms of vaccine-induced protection against covid-19 in humans. Nat Rev Immunol. (2021) 21:475–84. doi: 10.1038/s41577-021-00578-z PMC8246128 34211186

[20] Pinto LA Dillner J Beddows S Unger ER. Immunogenicity of hpv prophylactic vaccines: serology assays and their use in hpv vaccine evaluation and development. Vaccine. (2018) 36:4792–9. doi: 10.1016/j.vaccine.2017.11.089 PMC6050153 29361344

[21] Bournazos S Wang TT Dahan R Maamary J Ravetch JV. Signaling by antibodies: recent progress. Annu Rev Immunol. (2017) 35:285–311. doi: 10.1146/annurev-immunol-051116-052433 28446061 PMC5613280

[22] Dong C. Cytokine regulation and function in T cells. Annu Rev Immunol. (2021) 39:51–76. doi: 10.1146/annurev-immunol-061020-053702 33428453

[23] Al-Amrani S Al-Jabri Z Al-Zaabi A Alshekaili J Al-Khabori M. Proteomics: concepts and applications in human medicine. World J Biol Chem. (2021) 12:57–69. doi: 10.4331/wjbc.v12.i5.57 34630910 PMC8473418

[24] Wang Y Zhu Q Sun R Yi X Huang L Hu Y. Longitudinal proteomic investigation of covid-19 vaccination. Protein Cell. (2023) 14:668–82. doi: 10.1093/procel/pwad004 PMC10501184 36930526

[25] Kemp TJ Quesinberry JT Cherry J Lowy DR Pinto LA Miller MB. Selection, characterization, calibration, and distribution of the U.S. Serology standard for anti-sars-cov-2 antibody detection. J Clin Microbiol. (2022) 60:e0099522. doi: 10.1128/jcm.00995-22 36222529 PMC9667770

[26] Candia J Cheung F Kotliarov Y Fantoni G Sellers B Griesman T. Assessment of variability in the somascan assay. Sci Rep. (2017) 7:14248. doi: 10.1038/s41598-017-14755-5 29079756 PMC5660188

[27] NIBSC. Who international standard first who international standard for anti-sars-cov-2 immunoglobulin (Human) nibsc code: 20/136 instructions for use (Version 2.0, dated 17/12/2020). (2020) Medicines & Healthcare Products Regulatory Agency. Published by: National Institute for Biological Standards and Control, located at Potters Bar, Hertfordshire, EN6 3QG..

[28] Han K Singh K Rodman MJ Hassanzadeh S Baumer Y Huffstutler RD. Identification and validation of nutrient state-dependent serum protein mediators of human cd4(+) T cell responsiveness. Nutrients. (2021) 13:1-19. doi: 10.3390/nu13051492 PMC8146063 33924911

[29] Hwang M Assassi S Zheng JM Castillo J Chavez R Vanarsa K. Quantitative proteomic screening uncovers candidate diagnostic and monitoring serum biomarkers of ankylosing spondylitis. Arthritis Res Ther. (2023) 25:57. doi: 10.1186/s13075-023-03044-4 37041650 PMC10088143

[30] Liao YX Wang J Jaehnig EJ Shi ZA Zhang B. Webgestalt 2019: gene set analysis toolkit with revamped uis and apis. Nucleic Acids Res. (2019) 47:W199–205. doi: 10.1093/nar/gkz401 PMC6602449 31114916

[31] Croft D O’Kelly G Wu G Haw R Gillespie M Matthews L. Reactome: A database of reactions, pathways and biological processes. Nucleic Acids Res. (2011) 39:D691–7. doi: 10.1093/nar/gkq1018 PMC3013646 21067998

[32] Kanehisa M Goto S Furumichi M Tanabe M Hirakawa M. Kegg for representation and analysis of molecular networks involving diseases and drugs. Nucleic Acids Res. (2010) 38:D355–D60. doi: 10.1093/nar/gkp896 PMC2808910 19880382

[33] Wu Z Li T. Nanoparticle-mediated cytoplasmic delivery of messenger rna vaccines: challenges and future perspectives. Pharm Res. (2021) 38:473–8. doi: 10.1007/s11095-021-03015-x PMC7928182 33660201

[34] Rijkers GT Weterings N Obregon-Henao A Lepolder M Dutt TS van Overveld FJ. Antigen presentation of mrna-based and virus-vectored sars-cov-2 vaccines. Vaccines (Basel). (2021) 9:1-17. doi: 10.3390/vaccines9080848 PMC8402319 34451973

[35] Feng S Phillips DJ White T Sayal H Aley PK Bibi S. Correlates of protection against symptomatic and asymptomatic sars-cov-2 infection. Nat Med. (2021) 27:2032–40. doi: 10.1038/s41591-021-01540-1 PMC8604724 34588689

[36] Arunachalam PS Scott MKD Hagan T Li C Feng Y Wimmers F. Systems vaccinology of the bnt162b2 mrna vaccine in humans. Nature. (2021) 596:410–6. doi: 10.1038/s41586-021-03791-x PMC8761119 34252919

[37] Verbeke R Hogan MJ Lore K Pardi N. Innate immune mechanisms of mrna vaccines. Immunity. (2022) 55:1993–2005. doi: 10.1016/j.immuni.2022.10.014 36351374 PMC9641982

[38] Moghimi SM Simberg D. Pro-inflammatory concerns with lipid nanoparticles. Mol Ther. (2022) 30:2109–10. doi: 10.1016/j.ymthe.2022.04.011 PMC9047613 35487214

[39] Ndeupen S Qin Z Jacobsen S Bouteau A Estanbouli H Igyarto BZ. The mrna-lnp platform’s lipid nanoparticle component used in preclinical vaccine studies is highly inflammatory. iScience. (2021) 24:103479. doi: 10.1016/j.isci.2021.103479 34841223 PMC8604799

[40] Lonez C Bessodes M Scherman D Vandenbranden M Escriou V Ruysschaert JM. Cationic lipid nanocarriers activate toll-like receptor 2 and nlrp3 inflammasome pathways. Nanomed-Nanotechnol. (2014) 10:775–82. doi: 10.1016/j.nano.2013.12.003 24361386

[41] Tanaka T Legat A Adam E Steuve J Gatot JS Vandenbranden M. Dic14-amidine cationic liposomes stimulate myeloid dendritic cells through toll-like receptor 4. Eur J Immunol. (2008) 38:1351–7. doi: 10.1002/eji.200737998 18389479

[42] Uehata T Takeuchi O. Rna recognition and immunity-innate immune sensing and its posttranscriptional regulation mechanisms. Cells. (2020) 9:1-21. doi: 10.3390/cells9071701 PMC7407594 32708595

[43] Tokarz VL MacDonald PE Klip A. The cell biology of systemic insulin function. J Cell Biol. (2018) 217:2273–89. doi: 10.1083/jcb.201802095 PMC6028526 29622564

[44] Stoy J De Franco E Ye H Park SY Bell GI Hattersley AT. In celebration of a century with insulin - update of insulin gene mutations in diabetes. Mol Metab. (2021) 52:101280. doi: 10.1016/j.molmet.2021.101280 34174481 PMC8513141

[45] Liang TT Sang SY Shao Q Chen C Deng ZC Wang T. Abnormal expression and prognostic significance of epb41l1 in kidney renal clear cell carcinoma based on data mining. Cancer Cell Int. (2020) 20:356. doi: 10.1186/s12935-020-01449-8 32760223 PMC7393885

[46] Yuan X Piao L Wang L Han X Tong L Shao S. Erythrocyte membrane protein band 4.1-like 3 inhibits osteosarcoma cell invasion through regulation of snai1-induced epithelial-to-mesenchymal transition. Aging (Albany NY). (2020) 13:1947–61. doi: 10.18632/aging.202158 PMC7880352 33323539

[47] Cieślewicz A Dudek M Krela-Kaźmierczak I Jabłecka A Lesiak M Korzeniowska K. Pancreatic injury after covid-19 vaccine—a case report. Vaccines. (2021) 9:576. doi: 10.3390/vaccines9060576 34205898 PMC8228266

[48] Ozaka S Kodera T Ariki S Kobayashi T Murakami K. Acute pancreatitis soon after covid-19 vaccination: A case report. Med (Baltimore). (2022) 101:e28471. doi: 10.1097/md.0000000000028471 PMC8757977 35029194

[49] Parkash O Sharko A Farooqi A Ying GW Sura P. Acute pancreatitis: A possible side effect of covid-19 vaccine. Cureus. (2021) 13:e14741. doi: 10.7759/cureus.14741 34084669 PMC8163516

[50] Sasaki H Itoh A Watanabe Y Nakajima Y Saisho Y Irie J. Newly developed Type 1 diabetes after coronavirus disease 2019 vaccination: A case report. J Diabetes Investig. (2022) 13:1105–8. doi: 10.1111/jdi.13757 PMC9153841 35088548

[51] Cacdac R Jamali A Jamali R Nemovi K Vosoughi K Bayraktutar Z. Acute pancreatitis as an adverse effect of covid-19 vaccination. SAGE Open Med Case Rep. (2022) 10:2050313x221131169. doi: 10.1177/2050313x221131169 PMC9608244 36313269

[52] Bonora E Rizzi C Lesi C Berra P Coscelli C Butturini U. Insulin and C-peptide plasma levels in patients with severe chronic pancreatitis and fasting normoglycemia. Dig Dis Sci. (1988) 33:732–6. doi: 10.1007/bf01540438 3286157

[53] Ohneda A Kai Y Ishii S Matsuda K Horigome K. Insulin and glucagon response in patients with chronic pancreatitis. Tohoku J Exp Med. (1976) 120:287–98. doi: 10.1620/tjem.120.287 996852

[54] Perra L Balloy V Foussigniere T Moissenet D Petat H Mungrue IN. Chac1 is differentially expressed in normal and cystic fibrosis bronchial epithelial cells and regulates the inflammatory response induced by pseudomonas aeruginosa. Front Immunol. (2018) 9:2823. doi: 10.3389/fimmu.2018.02823 30555487 PMC6282009

[55] He S Zhang M Ye Y Zhuang J Ma X Song Y. Chac glutathione specific gamma-glutamylcyclotransferase 1 inhibits cell viability and increases the sensitivity of prostate cancer cells to docetaxel by inducing endoplasmic reticulum stress and ferroptosis. Exp Ther Med. (2021) 22:997. doi: 10.3892/etm.2021.10429 34345279 PMC8311285

[56] Yoshida N Abe H Ohkuri T Wakita D Sato M Noguchi D. Expression of the mage-A4 and ny-eso-1 cancer-testis antigens and T cell infiltration in non-small cell lung carcinoma and their prognostic significance. Int J Oncol. (2006) 28:1089–98. doi: 10.3892/ijo.28.5.1089 16596224

[57] Manohar S Jacob S Wang J Wiechecki KA Koh HWL Simoes V. Polyubiquitin chains linked by lysine residue 48 (K48) selectively target oxidized proteins. Antioxidants Redox Signaling. (2019) 31:1133–49. doi: 10.1089/ars.2019.7826 PMC6798811 31482721

[58] Mallette FA Richard S. K48-linked ubiquitination and protein degradation regulate 53bp1 recruitment at DNA damage sites. Cell Res. (2012) 22:1221–3. doi: 10.1038/cr.2012.58 PMC3411168 22491476

[59] Hussain T Tan B Yin Y Blachier F Tossou MC Rahu N. Oxidative stress and inflammation: what polyphenols can do for us? Oxid Med Cell Longev. (2016) 2016:7432797. doi: 10.1155/2016/7432797 27738491 PMC5055983

[60] Georgieva E Ananiev J Yovchev Y Arabadzhiev G Abrashev H Abrasheva D. Covid-19 complications: oxidative stress, inflammation, and mitochondrial and endothelial dysfunction. Int J Mol Sci. (2023) 24:14876. doi: 10.3390/ijms241914876 37834324 PMC10573237

[61] Dornbush S Aeddula NR. Physiology, leptin. In: Statpearls. StatPearls Publishing, Treasure Island (FL (2024). 30725723

[62] Tsuchiya H Fujio K. Emerging role of leptin in joint inflammation and destruction. Immunol Med. (2022) 45:27–34. doi: 10.1080/25785826.2021.1948689 34362290

[63] Larsson A Lipcsey M Hultström M Frithiof R Eriksson M. Plasma leptin is increased in intensive care patients with covid-19—an investigation performed in the pronmed-cohort. Biomedicines. (2022) 10:4. doi: 10.3390/biomedicines10010004 PMC8773415 35052684

[64] Magister S Kos J. Cystatins in immune system. J Cancer. (2013) 4:45–56. doi: 10.7150/jca.5044 23386904 PMC3564246

[65] Hill LJ Di Pietro V Hazeldine J Davies D Toman E Logan A. Cystatin D (Cst5): an ultra-early inflammatory biomarker of traumatic brain injury. Sci Rep. (2017) 7:5002. doi: 10.1038/s41598-017-04722-5 28694499 PMC5504020

[66] Collins AR Grubb A. Cystatin D, a natural salivary cysteine protease inhibitor, inhibits coronavirus replication at its physiologic concentration. Oral Microbiol Immunol. (1998) 13:59–61. doi: 10.1111/j.1399-302x.1998.tb00753.x 9573825 PMC7167923

[67] Nocera AL Mueller SK Workman AD Wu D McDonnell K Sadow PM. Cystatin sn is a potent upstream initiator of epithelial-derived type 2 inflammation in chronic rhinosinusitis. J Allergy Clin Immunol. (2022) 150:872–81. doi: 10.1016/j.jaci.2022.04.034 PMC9547833 35660375

[68] Oshiumi H Matsumoto M Funami K Akazawa T Seya T. Ticam-1, an adaptor molecule that participates in toll-like receptor 3-mediated interferon-beta induction. Nat Immunol. (2003) 4:161–7. doi: 10.1038/ni886 12539043

[69] Zhu G Chen X Wang X Li X Du Q Hong H. Expression of the rip-1 gene and its role in growth and invasion of human gallbladder carcinoma. Cell Physiol Biochem. (2014) 34:1152–65. doi: 10.1159/000366328 25277242

[70] Festjens N Vanden Berghe T Cornelis S Vandenabeele P. Rip1, a kinase on the crossroads of a cell’s decision to live or die. Cell Death Differentiation. (2007) 14:400–10. doi: 10.1038/sj.cdd.4402085 17301840

[71] Li WJ Yuan JY. Targeting ripk1 kinase for modulating inflammation in human diseases. Front Immunol. (2023) 14:1159743. doi: 10.3389/fimmu.2023.1159743 36969188 PMC10030951

[72] Mifflin L Ofengeim D Yuan JY. Receptor-interacting protein kinase 1 (Ripk1) as a therapeutic target. Nat Rev Drug Discovery. (2020) 19:553–71. doi: 10.1038/s41573-020-0071-y PMC7362612 32669658

[73] Cusson-Hermance N Khurana S Lee TH Fitzgerald KA Kelliher MA. Rip1 mediates the trif-dependent toll-like receptor 3- and 4-induced nf-kappab activation but does not contribute to interferon regulatory factor 3 activation. J Biol Chem. (2005) 280:36560–6. doi: 10.1074/jbc.M506831200 16115877

[74] Cui J Chen Y Wang HY Wang RF. Mechanisms and pathways of innate immune activation and regulation in health and cancer. Hum Vaccin Immunother. (2014) 10:3270–85. doi: 10.4161/21645515.2014.979640 PMC4514086 25625930

[75] Aid M Stephenson KE Collier AY Nkolola JP Michael JV McKenzie SE. Activation of coagulation and proinflammatory pathways in thrombosis with thrombocytopenia syndrome and following covid-19 vaccination. Nat Commun. (2023) 14:6703. doi: 10.1038/s41467-023-42559-x 37872311 PMC10593859

[76] Bekal S Husari G Okura M Huang CA Bukari MS. Thrombosis development after mrna covid-19 vaccine administration: A case series. Cureus. (2023) 15:e41371. doi: 10.7759/cureus.41371 37546104 PMC10400017

[77] Bilotta C Perrone G Adelfio V Spatola GF Uzzo ML Argo A. Covid-19 vaccine-related thrombosis: A systematic review and exploratory analysis. Front Immunol. (2021) 12:729251. doi: 10.3389/fimmu.2021.729251 34912330 PMC8666479

[78] Leung W-Y Wu HHL Floyd L Ponnusamy A Chinnadurai R. Covid-19 infection and vaccination and its relation to amyloidosis: what do we know currently? Vaccines. (2023) 11:1-14. doi: 10.3390/vaccines11071139 PMC10383215 37514955

[79] Pan JS Hong MZ Ren JL. Reactive oxygen species: A double-edged sword in oncogenesis. World J Gastroenterol. (2009) 15:1702–7. doi: 10.3748/wjg.15.1702 PMC2668775 19360913

[80] Lee S Rauch J Kolch W. Targeting mapk signaling in cancer: mechanisms of drug resistance and sensitivity. Int J Mol Sci. (2020) 21:1-29. doi: 10.3390/ijms21031102 PMC7037308 32046099

[81] Batra R Whalen W Alvarez-Mulett S Gomez-Escobar LG Hoffman KL Simmons W. Multi-omic comparative analysis of covid-19 and bacterial sepsis-induced ards. PloS Pathog. (2022) 18:e1010819. doi: 10.1371/journal.ppat.1010819 36121875 PMC9484674

[82] Bouhaddou M Memon D Meyer B White KM Rezelj VV Correa Marrero M. The global phosphorylation landscape of sars-cov-2 infection. Cell. (2020) 182:685–712.e19. doi: 10.1016/j.cell.2020.06.034 32645325 PMC7321036

[83] Leone P Shin EC Perosa F Vacca A Dammacco F Racanelli V. Mhc class I antigen processing and presenting machinery: organization, function, and defects in tumor cells. J Natl Cancer Inst. (2013) 105:1172–87. doi: 10.1093/jnci/djt184 23852952

[84] Zhang Z Mateus J Coelho CH Dan JM Moderbacher CR Gálvez RI. Humoral and cellular immune memory to four covid-19 vaccines. Cell. (2022) 185:2434–51.e17. doi: 10.1016/j.cell.2022.05.022 35764089 PMC9135677

[85] Chow KT Gale M Jr. Loo YM. Rig-I and other rna sensors in antiviral immunity. Annu Rev Immunol. (2018) 36:667–94. doi: 10.1146/annurev-immunol-042617-053309 29677479

[86] Rehwinkel J Gack MU. Rig-I-like receptors: their regulation and roles in rna sensing. Nat Rev Immunol. (2020) 20:537–51. doi: 10.1038/s41577-020-0288-3 PMC7094958 32203325

[87] Aggarwal BB. Signaling pathways of the tnf superfamily: A double-edged sword. Nat Rev Immunol. (2003) 3:745–56. doi: 10.1038/nri1184 12949498

[88] Markiewski MM Lambris JD. The role of complement in inflammatory diseases from behind the scenes into the spotlight. Am J Pathol. (2007) 171:715–27. doi: 10.2353/ajpath.2007.070166 PMC1959484 17640961

[89] Zenobia C Hajishengallis G. Basic biology and role of interleukin-17 in immunity and inflammation. Periodontol 2000. (2015) 69:142–59. doi: 10.1111/prd.12083 PMC4530463 26252407

