# Supplementary material for: Proteomic and serologic assessments of responses to mRNA-1273 and BNT162b2 vaccines in human recipient sera
Source: Front Immunol. 2025 Jan 27;15:1502458. doi: 10.3389/fimmu.2024.1502458 (PMC11808009; doi:10.3389/fimmu.2024.1502458)
Supplement: Supplementary file 1 [file Table1.docx]

Supplementary Material

# Supplementary Tables

## Supplementary Table S1A Downregulated Proteomics Markers in Sera from Vaccine Recipients after Homologous Boost

| Male Recipients | | | | Female Recipients | | | |
| --- | --- | --- | --- | --- | --- | --- | --- |
| 1-Month | | 6-Months | | 1-Month | | 6-Months | |
| BNT 162b2 | mRNA-1273 | BNT 162b2 | mRNA-1273 | BNT 162b2 | mRNA-1273 | BNT 162b2 | mRNA-1273 |
| AAGAB  ABLIM3  ACYP2  AGFG1  AIDA  ALDH1A3  AP1G2  AP2B1  ARF4  ARFGAP2  ARHGDIB  ARL3  ARL8A  ARPC1B  BAG6  BIN2  BTK  BZW2  CA13  CACYBP  CALD1  CAMK2B  CAPZA1  CCM2  CD2AP  CDKN2D  CERT1  CES3  CHMP1A  CHMP2A  CLINT1  CRKL  CS  CSK  CSRP1  CTDSP1  CTDSPL  CXCL8  DAB2  DAPP1  DARS1  DARS2  DBI  DBNL  DNAJC16  DOK2  DPP7  DSG3  DTD1  DUSP28  DUSP3  DYNLL2  EEF1A1  EEF1D  EHD1  EHD3  EIF1  EIF1AY  EIF3B  EIF3G  EIF4B  EIF4G2  EIF4H  EIF5A2  EPN1  EPS15L1  FAF2  FER  FERMT3  FGF19  FHIP2A  FKBP1B  FKBP3  FLI1  FYN  GABARAPL2  GGA1  GGA3  GMFB  GMFG  GP6  GRAP2  GRB10  GRB2  GUK1  HGS  HNRNPDL  HNRNPF  HOMER1  ILK  IMPACT  IRF3  ITGA2B\|ITGB3  KPNA5  LACTB2  LGALSL  LGMN  LONP1  LPIN1  LRRFIP2  LTB4R  MANF  MAP1LC3B  MAP4K1  MAPRE1  MAPRE3  MBNL1  MBNL2  MDP1  ME2  MESD  MGMT  MOB1B  MPIG6B  MTHFS  MVB12B  NARS1  NCK2  NCKIPSD  NEDD8  NPLOC4  NSFL1C  NUB1  NUDT2  OPHN1  OVCA2  PACSIN2  PAGR1  PCBP1  PDIA5  PDLIM1  PEAR1  PFN1  PIH1D1  PIN4  PLEK  PPIA  PPIF  PPIH  PPP1R9B  RAB11A  RAB11B  RAB1A  RAB1B  RAB21  RAB27B  RAB3C  RAB6A  RAB6B  RAB7A  RAD23B  RBMS1  RBPMS2  RGS10  RTP4  SH3BGRL2  SH3BGRL3  SH3GL2  SH3GL3  SH3PXD2B  SHANK3  SHARPIN  SHC1  SLC9A3R1  SMAD2  SMAD5  SMAP1  SMTN  SNX12  SPAST  SSMEM1  STIM1  STX12  STX4  STX8  SUGT1  SUMO2  SUMO3  SYAP1  SYTL4  TAGLN2  TBCB  TBCE  TFCP2L1  TIA1  TMOD2  TMOD3  TOLLIP  TPM4  TPT1  TRAPPC2  TRIP10  TSG101  TSTD1  TWF2  TXNDC12  TXNL4B  UBE2D4  UBE2I  UBE2K  UBE2N  UBE2N\|UBE2V1  UBE2N\|UBE2V2  UBE2V1  UBL4A  UBL5  UFM1  USP8  VAPA  VASP  VAV1  VCPIP1  VPS4A  VTA1  VTI1B  WAS  ZFAND1  ZFYVE27 | B2M  CKB\|CKM  CKM  CRISPLD2  CTNNBIP1  CXCL8  IL36A  KLK12  LTBR  LTF  PGLYRP1  RAD51  TPM2  ZAP70 | A2M  AAGAB  ABLIM3  ACP2  ACYP2  AGFG1  AKT3  AP1G2  AP2B1  ARF3  ARFGAP2  ARL3  ARPC1B  ATOX1  ATP5PB  BAG6  BCL2L1  BIN2  BTK  C11orf68  C1orf162  C3  C8A\|C8B\|C8G  CA13  CACYBP  CALD1  CAMK2B  CAPZA1  CCM2  CD2AP  CDKN2D  CERT1  CFL2  CHMP1A  CLINT1  CRISPLD2  CRKL  CSK  CSRP1  CTDSP1  CTDSPL  CTNNBIP1  CX3CL1  CXCL8  DAB2  DAPP1  DBI  DCP1B  DDX19B  DNAJC16  DNM1  DOK2  DTX3L  DUSP28  DUSP3  DYNLRB1  EEF1A1  EEF1AKMT1  EIF1  EIF3B  EIF3G  EIF4B  EIF4G2  EIF4H  ENPEP  EPN1  EPS15L1  FAF2  FER  FERMT3  FHIP2A  FKBP1B  FKBP3  FLI1  FTH1\|FTL  FTL  FXYD6  GABARAPL2  GALK1  GGA1  GGA3  GMFB  GMFG  GRAP2  GRB2  GSK3B  HAMP  HGS  HOMER1  IL36A  IL37  ILK  IMPACT  IRF3  KIT  KLK12  KNG1  LACTB2  LDHA  LGALSL  LONP1  LPIN1  LRRFIP2  LTBR  MANF  MAP1LC3B  MAP4K1  MAPKAPK5  MAPRE1  MBNL1  MBNL2  MDP1  MESD  MGMT  MMP8  MMP9  MOB1B  MPIG6B  MSI2  MTHFS  MVB12B  MVP  NARS1  NCK2  NCKIPSD  NEDD8  NIF3L1  NPLOC4  NRGN  NSFL1C  NUB1  NUDT2  OPHN1  OVCA2  PAK3  PDE5A  PDLIM1  PFKFB1  PFN1  PIH1D1  PPIH  PPP1R9B  PTPN11  PUS7  RAB18  RAB1A  RAB27B  RAB6A  RAB6B  RACGAP1  RAD23B  RAD51  RBMS1  RBPMS2  RGS10  RTP4  SAR1B  SCP2  SERPINB1  SH3BGRL2  SH3BGRL3  SH3GL2  SH3GL3  SHANK3  SHARPIN  SHC1  SMAD2  SMAD5  SMAP1  SMTN  SNX12  SPAST  SSMEM1  ST8SIA4  STX12  STX4  STX8  SUGT1  SUMO2  SUMO3  SYAP1  TAGLN2  TBCB  TBCE  TFCP2L1  TG  TIA1  TMOD2  TMOD3  TOLLIP  TPM2  TPT1  TRAPPC2  TRIP10  TSG101  TSTD1  TTC27  TWF2  TXNDC12  TXNDC9  TXNL4B  UBE2D4  UBE2K  UBE2M  UBL4A  UBL5  UBQLN2  UBQLN4  UFM1  USP8  VAPA  VAV1  VCPIP1  VPS4A  VTA1  VTI1B  ZAP70  ZC3H12A  ZFAND1  ZNF276 | A2M  APOB  ATP5PB  C3  C4A\|C4B  CCL28  CFL2  CRISPLD2  CXCL3  DCP1B  FXYD6  IL36A  KNG1  LDHA  MAPKAPK5  MB  METTL2B  MMP9  MYBPC1  NIF3L1  OASL  PFKFB1  PROK2  PUS7  RACGAP1  ST8SIA4  STK3  TG | AAGAB  AAMDC  ABLIM3  ACOT13  ACP1  ACTN1  ACYP1  ACYP2  AFAP1L2  AGFG1  AIDA  AK1  ALDH1A3  ANXA11  AP1G2  AP2A2  AP2B1  ARF3  ARF4  ARFGAP2  ARHGAP25  ARHGAP45  ARHGDIB  ARL3  ARPC1B  ARRB1  ATOX1  ATP6V1C1  ATP6V1C2  B2M  BAG6  BIN2  BPI  BTK  BZW2  C11orf68  CA13  CAB39  CACYBP  CALD1  CAMK2B  CAMK2D  CAP1  CAPZA1  CCM2  CD2AP  CD84  CDKN2D  CERT1  CES3  CHMP1A  CHMP2A  CHMP3  CLINT1  CNOT1  COL6A3  COMMD10  CRADD  CRKL  CS  CSK  CSRP1  CTDSP1  CTDSPL  CTNNBIP1  CTSC  CXCL8  CYB5R4  DAB2  DAPP1  DARS1  DARS2  DBI  DBNL  DCTD  DDX19A  DDX19B  DNAJB8  DNAJC16  DNAJC5B  DNM1  DOK2  DPP7  DSG3  DSTN  DTD1  DTX3L  DUSP28  DUSP3  DYNLL2  DYNLRB1  EDAR  EEF1A1  EEF1D  EGF  EHD1  EHD3  EIF1  EIF1AY  EIF3B  EIF3G  EIF4A1  EIF4A2  EIF4B  EIF4G2  EIF4H  EIF5  EIF5A2  EPN1  EPS15L1  FAF2  FBLN5  FER  FERMT3  FHIP2A  FHIT  FKBP1A  FKBP1B  FKBP3  FKBP5  FLI1  FLII  FLNA  FYN  G3BP1  GABARAPL2  GGA1  GGA3  GIPC1  GLB1  GLRX3  GMFB  GMFG  GNPNAT1  GP6  GRAP2  GRB10  GRB2  GUK1  H1.2  HCLS1  HGS  HNRNPDL  HNRNPF  HNRNPK  HOMER1  HOMER2  IDI1  IL18  ILK  IMPACT  INPP5A  IRF3  IST1  KPNA5  KRAS  LACTB2  LGALSL  LGMN  LONP1  LPIN1  LRRFIP2  LRSAM1  LSM12  LTB4R  LTF  LYN  MAGEA4  MANF  MAP1LC3B  MAP4K1  MAPRE1  MAPRE3  MBNL1  MBNL2  MDH2  MDP1  ME2  MESD  MGMT  MLEC  MOB1B  MPIG6B  MPO  MSI2  MTHFS  MVB12B  MYCBP  MYL12A  MYL9  NAA50  NAPA  NARS1  NCK2  NCKIPSD  NECAP2  NEDD8  NPLOC4  NRAS  NSFL1C  NT5C3A  NUB1  NUDT2  NUDT3  OLR1  OPHN1  OSBPL1A  OVCA2  OXSR1  PACSIN2  PAGR1  PARK7  PCBP1  PDIA6  PDLIM1  PDPK1  PEAR1  PEBP1  PFN1  PGLS  PIH1D1  PIN1  PIN4  PKP2  PLEK  PLPBP  POMK  PPFIA1  PPIA  PPIF  PPIH  PPP1R9B  PRKAR2B  PRTN3  PTP4A2  PTPN11  PTPN6  PTRHD1  PUDP  PYGB  RAB11A  RAB11B  RAB18  RAB1A  RAB1B  RAB21  RAB27B  RAB31  RAB33A  RAB3A  RAB5B  RAB5C  RAB6A  RAB6B  RAB7A  RAD23B  RBM19  RBMS1  RBPMS2  RGS10  RNASE3  RTP4  RWDD4  SCP2  SERPINB1  SERPINB9  SFTPD  SH3BGRL2  SH3BGRL3  SH3GL2  SH3GL3  SH3PXD2B  SHANK3  SHARPIN  SHC1  SHMT2  SLBP  SLC9A3R1  SLMAP  SMAD2  SMAD5  SMAP1  SMTN  SNX11  SNX12  SPART  SPAST  SSMEM1  STIM1  STK10  STX12  STX4  STX8  SUGT1  SUMO2  SUMO3  SUMO4  SYAP1  SYNE2  SYTL4  TAGLN2  TAX1BP3  TBCB  TBCE  TCN1  TEC  TFCP2L1  THBS1  TIA1  TMOD2  TMOD3  TOLLIP  TPM4  TPT1  TRAPPC2  TREML1  TRIP10  TRMT6  TSG101  TSTD1  TTC27  TWF2  TXNDC12  TXNDC9  TXNL4B  UBA7  UBASH3B  UBE2D4  UBE2E1  UBE2I  UBE2K  UBE2M  UBE2N  UBE2N\|UBE2V1  UBE2N\|UBE2V2  UBE2V1  UBE2V2  UBE3A  UBL4A  UBL5  UBQLN2  UBQLN4  UFM1  USP15  USP8  VAPA  VASP  VAV1  VCPIP1  VPS26A  VPS4A  VSIR  VTA1  VTI1A  VTI1B  WAS  YWHAZ  ZC3H12A  ZFAND1  ZFYVE27  ZRANB1 | AAGAB  AAMDC  ABLIM3  ACADVL  ACAP2  ACBD6  ACOT13  ACP1  ACTN1  ACYP1  ACYP2  AGFG1  AGFG2  AHSA1  AIDA  AK1  AK2  AKT3  ANXA11  AP1G2  AP2A2  AP2B1  ARF3  ARF4  ARFGAP2  ARFIP1  ARHGAP25  ARHGAP45  ARHGDIB  ARID3C  ARL3  ARL8A  ARPC1B  ARPC3  ARRB1  ASPSCR1  ATOX1  ATP5PO  ATP6V1C1  ATP6V1C2  B2M  BAG6  BID  BIN2  BTK  BZW2  C11orf68  C4A\|C4B  CA13  CAB39  CACYBP  CALD1  CAMK2A  CAMK2B  CAMK2D  CAP1  CCL5  CCM2  CD2AP  CD84  CDKN2D  CEACAM8  CERT1  CES3  CHAC2  CHMP1A  CHMP2A  CHMP3  CIRBP  CLEC1B  CLIC1  CLIC4  CLINT1  CMPK1  CNOT1  COL6A3  COMMD10  COPB2  CRADD  CRK  CRKL  CRYBB1  CS  CSK  CSNK1G1  CSRP1  CSRP3  CTDSP1  CTDSPL  CTNNBIP1  CXCL3  CXCL8  CYB5R4  DAB2  DAPP1  DARS1  DARS2  DBI  DBNL  DCTD  DDX19A  DDX19B  DDX6  DECR1  DENR  DLG2  DNAJB8  DNAJC16  DNAJC5B  DNM1  DOK2  DPP7  DSG3  DSTN  DTD1  DTX3L  DUSP28  DUSP3  DYNLL2  DYNLRB1  EEF1A1  EEF1D  EGF  EHD1  EHD3  EIF1  EIF1AY  EIF1B  EIF2S2  EIF3B  EIF3G  EIF4A1  EIF4A2  EIF4B  EIF4E  EIF4G2  EIF4H  EIF5  EIF5A2  ENAH  EPN1  EPS15L1  FAF2  FBLN5  FER  FERMT3  FEV  FHIP2A  FHIT  FHOD1  FKBP1A  FKBP1B  FKBP2  FKBP3  FKBP5  FKBP7  FLI1  FLII  FLNA  FMR1  FUBP1  FXR1  FYN  G3BP1  GABARAPL2  GAGE2D  GGA1  GGA3  GIPC1  GLRX3  GMFB  GMFG  GNPNAT1  GOLPH3L  GP6  GRAP2  GRB10  GRB2  GUK1  HAMP  HCLS1  HGS  HIBCH  HNRNPDL  HNRNPF  HNRNPK  HOMER1  HOMER2  HS2ST1  HSPE1  IDI1  ILK  IMPACT  INPP5A  INPP5B  IRAG1  IRF3  ISG15  IST1  ITGA2B\|ITGB3  ITGB1BP2  IVD  KPNA5  KRAS  LACTB2  LCN2  LGALSL  LGMN  LONP1  LPIN1  LRRC59  LRRFIP2  LRSAM1  LTB4R  LTF  LYN  MANF  MAP1LC3A  MAP1LC3B  MAP4K1  MAPRE1  MAPRE3  MBNL1  MBNL2  MDH2  MDP1  ME2  MESD  MGMT  MLEC  MMP8  MOB1B  MPIG6B  MPO  MRPL1  MRPL12  MSI2  MTHFS  MTIF3  MTSS2  MVB12B  MYCBP  MYL12A  MYL9  MYO6  NAA50  NAPA  NARS1  NCK2  NCKIPSD  NECAP2  NEDD8  NEK7  NME7  NPLOC4  NRAS  NSFL1C  NUB1  NUDC  NUDT2  NUDT21  NUDT3  OLR1  OPHN1  OSBPL11  OSBPL1A  OVCA2  OXSR1  PABPC4  PACSIN2  PAGR1  PAPOLG  PARK7  PCBP1  PCBP2  PDCD5  PDE5A  PDIA5  PDIA6  PDLIM1  PDPK1  PEAR1  PEBP1  PFN1  PGLS  PGLYRP1  PIH1D1  PIN1  PIN4  PKP2  PLA2G12A  PLEK  PLEKHA7  PLPBP  PNPT1  POLR2M  PPFIA1  PPIA  PPIF  PPIH  PPIL1  PPP1CC  PPP1R9B  PRKAA2\|PRKAB2\|PRKAG1  PRKACA  PRKAR1A  PRKAR2B  PRKCA  PRTN3  PTP4A2  PTPN11  PTPN6  PTRHD1  PUDP  PYGB  RAB11A  RAB11B  RAB14  RAB18  RAB1A  RAB1B  RAB21  RAB24  RAB27B  RAB2A  RAB31  RAB32  RAB33A  RAB3A  RAB3C  RAB3D  RAB4B  RAB5A  RAB5B  RAB5C  RAB6A  RAB6B  RAB7A  RAB8B  RAC1  RAC2  RAD23A  RAD23B  RANBP3  RBM19  RBMS1  RBPMS2  RGS10  RGS18  RNASE3  RNF114  ROCK2  RTP4  RWDD4  SCARF1  SCP2  SEC22A  SELENOW  SELP  SERPINB1  SERPINB9  SGTA  SH3BGRL  SH3BGRL2  SH3BGRL3  SH3GL2  SH3GL3  SH3PXD2B  SHANK3  SHARPIN  SHC1  SHMT2  SLBP  SLC9A3R1  SLMAP  SMAD2  SMAD3  SMAD5  SMAP1  SMTN  SNRPG  SNTB1  SNX11  SNX12  SNX4  SPART  SPAST  SRC  SSMEM1  STIM1  STK24  STX12  STX4  STX8  SUGT1  SUMO2  SUMO3  SUMO4  SYAP1  SYNE2  SYTL4  TAGLN2  TAX1BP3  TBCB  TBCE  TEC  TFCP2L1  TIA1  TIAL1  TIPRL  TMOD2  TMOD3  TNFSF14  TOLLIP  TPM4  TPT1  TRAPPC2  TRIP10  TRMT6  TSG101  TSTD1  TTC1  TTC27  TTC9  TWF1  TWF2  TXNDC12  TXNDC9  TXNL4B  TYMP  UBA7  UBASH3B  UBE2D4  UBE2E1  UBE2I  UBE2K  UBE2M  UBE2N  UBE2N\|UBE2V1  UBE2N\|UBE2V2  UBE2V1  UBE2V2  UBE3A  UBL4A  UBL5  UBQLN2  UBQLN3  UBQLN4  UCN3  UFC1  UFD1  UFM1  USP15  USP22  USP8  VAPA  VASP  VAV1  VCPIP1  VIL1  VPS26A  VPS4A  VSIR  VTA1  VTI1A  VTI1B  WAS  YWHAH  YWHAZ  ZC3H12A  ZFAND1 | A2M  AAGAB  ABLIM3  ACAP2  ACP2  ACYP2  AFAP1L2  AGFG1  AP1G2  AP2B1  ARF3  ARF4  ARFGAP2  ARL3  ARPC1B  ATOX1  ATP5PB  ATP6V1F  BAG6  BIN2  BTK  C3  CA13  CACYBP  CALD1  CAMK2B  CAPZA1  CCM2  CD2AP  CD84  CDKN2D  CERT1  CES3  CFL2  CLINT1  COL6A1  COMMD10  CRKL  CRYBA2  CSRP1  CTDSP1  CTDSPL  CTNNBIP1  CTSC  CXCL2  CXCL3  CXCL8  DAB2  DAPP1  DCP1B  DDX19A  DDX19B  DNAJC5B  DNM1  DOK2  DTX3L  DUSP28  DUSP3  DYNLL2  ECHS1  EDAR  EEF1AKMT1  EHD3  EIF1  EIF3B  EIF3G  EIF4B  EIF4H  ENPEP  EPN1  EPS15L1  F2  FAF2  FAIM  FER  FERMT3  FEV  FGL1  FHIP2A  FKBP1A  FKBP1B  FKBP2  FKBP3  FTMT  FXYD6  FYN  G3BP1  GABARAPL2  GGA1  GGA3  GLB1  GLIPR2  GMFB  GMFG  GP6  GRAP2  GRB2  HGS  HOMER1  HOMER2  IL18  IL36A  ILK  IMPACT  KNG1  LDHA  LGALSL  LONP1  LPIN1  LRRFIP2  MAGEA4  MANF  MAP1LC3A  MAP1LC3B  MAP4K1  MAPRE1  MBNL1  MBNL2  MDP1  MESD  MGMT  MOB1B  MPIG6B  MSI2  MVB12B  MVP  NARS1  NCK2  NCKIPSD  NEDD8  NIF3L1  NPLOC4  NSFL1C  NUB1  NUDT2  OASL  OPHN1  OVCA2  PAGR1  PAPOLG  PDLIM1  PDPK1  PEAR1  PFKFB1  PPIH  PPP1R9B  PTP4A2  PTPN11  PUDP  PUS7  PYGB  RAB11A  RAB11B  RAB18  RAB1A  RAB27B  RAB33A  RAB6A  RAB6B  RACGAP1  RAD23B  RANBP3  RBMS1  RBPMS2  RGS10  RTP4  SCP2  SEC22A  SERPINB1  SERPING1  SH3BGRL2  SH3BGRL3  SHANK3  SHARPIN  SHC1  SLMAP  SMAP1  SMTN  SNX12  SPART  SPAST  SSMEM1  ST8SIA4  STX12  STX4  STX8  SUGT1  SUMO2  SUMO3  SUMO4  SYAP1  TAGLN2  TAX1BP3  TBCE  TFCP2L1  TG  THOP1  TIA1  TKFC  TMOD2  TMOD3  TOLLIP  TRAPPC2  TRIP10  TSG101  TTC27  TWF2  TXNDC12  TXNDC9  TXNL4B  UBE2D4  UBE2E1  UBE2M  UBE3A  UBL4A  UBL5  UBQLN2  UBQLN4  UFM1  USP8  VAPA  VASP  VCPIP1  VPS4A  VSIR  VTA1  VTI1B  ZC3H12A  ZFAND1 | AAGAB  AAMDC  ABLIM3  ACAP2  ACYP2  AGFG1  AHSA1  AP1G2  AP2A2  AP2B1  ARF3  ARF4  ARFGAP2  ARL3  B2M  BAG6  BIN2  BTK  C11orf68  C3  CA13  CA4  CACYBP  CALD1  CAMK2B  CCM2  CD2AP  CDKN2D  CERT1  CES3  CFL2  CHMP2A  CLINT1  CNOT1  COMMD10  CRISPLD2  CRKL  CSK  CSRP1  CTDSP1  CTDSPL  CTNNBIP1  CXCL2  CXCL3  CXCL8  CYB5R4  DAB2  DAPP1  DARS1  DBI  DCP1B  DCTD  DDX19B  DNAJC16  DNM1  DOK2  DTX3L  DUSP28  DUSP3  DYNLL2  DYNLRB1  EEF1A1  EEF1D  EHD1  EHD3  EIF3B  EIF3G  EIF4B  EIF4G2  EIF4H  EIF5A2  ENO2  EPN1  EPS15L1  FAF2  FER  FERMT3  FGF19  FHIP2A  FHIT  FKBP1B  FKBP3  FKBP5  FLI1  FLII  FMR1  FTH1\|FTL  FTL  FXYD6  FYN  G3BP1  GABARAPL2  GGA1  GGA3  GMFB  GMFG  GNPNAT1  GOLPH3L  GP6  GRAP2  GRB10  GRB2  HAMP  HGS  HOMER1  HOMER2  HOXA11  HS2ST1  IDI1  IL36A  IMPACT  IRF3  IST1  KNG1  LACTB2  LGALSL  LONP1  LPIN1  LRRFIP2  LTB4R  MANF  MAP1LC3B  MAP4K1  MAPRE1  MAPRE3  MBNL1  MBNL2  MDP1  MESD  MGMT  MLEC  MOB1B  MPIG6B  MSI2  MTHFS  MVB12B  NARS1  NCK2  NCKIPSD  NEDD8  NIF3L1  NPLOC4  NSFL1C  NUB1  NUDT2  OPHN1  OVCA2  PABPC4  PDE5A  PDIA6  PDLIM1  PFKFB1  PFN1  PIH1D1  PIN4  PLEK  PPFIA1  PPIH  PPP1R9B  PTPN11  PTPN6  PUS7  RAB11A  RAB11B  RAB14  RAB18  RAB1A  RAB1B  RAB21  RAB27B  RAB33A  RAB3A  RAB6A  RAB6B  RAB7A  RAD23B  RANBP3  RBMS1  RBPMS2  RGS10  RPS10  RTP4  SCP2  SEC22A  SELENOW  SERPINB1  SERPINB9  SERPING1  SGTA  SH3BGRL2  SH3BGRL3  SH3GL2  SHANK3  SHARPIN  SHC1  SLC9A3R1  SLMAP  SMAD2  SMAD5  SMAP1  SMTN  SPART  SPAST  SSMEM1  ST8SIA4  STIM1  STX12  STX4  STX8  SUGT1  SUMO2  SUMO3  SYAP1  SYTL4  TAGLN2  TBCB  TBCE  TEC  TFCP2L1  TG  TIA1  TMOD2  TMOD3  TOLLIP  TPM4  TPT1  TRAPPC2  TRIP10  TSG101  TSTD1  TTC1  TTC27  TWF2  TXNDC12  TXNDC9  TXNL4B  TYMP  UBE2D4  UBE2E1  UBE2V2  UBE3A  UBL4A  UBQLN2  UBQLN4  UFM1  USP8  VAPA  VAV1  VCPIP1  VPS4A  VTA1  VTI1B  ZC3H12A  ZFAND1 |

**Supplementary Table S1B Upregulated Proteomics Markers in Sera from Vaccine Recipients after Homologous Boost**

| Male Recipients | | | | Female Recipients | | | |
| --- | --- | --- | --- | --- | --- | --- | --- |
| 1-Month | | 6-Months | | 1-Month | | 6-Months | |
| BNT 162b2 | mRNA-1273 | BNT 162b2 | mRNA-1273 | BNT 162b2 | mRNA-1273 | BNT162b2 | mRNA-1273 |
| ADH1C  ADH4  APOA5  CHAC1  CTAG1A\| CTAG1B  CYP2C19  FTCD  SORD  UBE2D1\| UBB | AGO2  BLVRB  EPB41L1  HMBS  IGL\|IGHE\| IGK  INS  PAFAH1B3  TBCEL  TMOD1  UBE2D1\| UBB  USP14 | ACAT1  ADSL  APOA5  ARG1  BLVRB  BPGM  CA1  CA3  CCDC50  CDH7  CHGA  CKB\|CKM  COQ9  CTAG1A\| CTAG1B  CXCL11  CXCL12  DDX39B  DEFA1  ECHDC1  EIF1B  EPB41  ERH  FGR  FN3K  FTCD  GAA  GAPDH  GMPS  H2AC1  H2AC11  H2AW  H2BC12  H2BC21  H3C1  HBEGF  HMBS  HMGCS1  HNRNPA0  HNRNPA2B1  INS  KPNB1  LANCL2  LDHA  LEAP2  LSP1  MAGOH  MEP1A  MFAP4  NCF1  NIT2  PA2G4  PABPN1  PAFAH1B3  PCBP2  PCNA  PGD  PKLR  PLS3  PNP  PRKCB  RAB8B  RPL26L1  RPL30  RPL5  RPS4X  RRM1  RTCA  S100A12  SCPEP1  SFTPD  SNRPB2  SPI1  SRGN  TBCEL  TCEA1  TFF1  TIMP3  TLR5  TMOD1  UBE2D1\|UBB  UBE2D3\|UBB  USP14  VWF | ABHD14A  ACP1  ADK  ADPRS  ADSL  AGO2  AK1  AKR7A2  ALAD  ALKAL2  ANXA1  APEX1  APRT  ARG1  ARHGAP25  ATP6V1C2  ATXN3  BAD  BAGE2  BLVRA  BLVRB  BPGM  CA1  CAP1  CAPG  CAT  CBL  CDK2  CFAP36  CGGBP1  CHGA  CHMP1B  CHMP2B  CHMP4A  CIAO1  CILP  CIRBP  CLIC2  CMPK1  CNRIP1  COPB2  COQ9  CPNE1  CPPED1  CSNK2A1\| CSNK2B  CTPS1  CXCL11  CXCL12  DBNL  DDI2  DDX39B  DDX6  DENND10  DNM2  DPCD  DTYMK  EEF1A1  EFHD2  EGF  EIF1AY  EIF1B  EIF2B1  EIF2S1  EIF2S2  EIF3M  EIF4A2  EIF4E  EIF5A  EPB41  EPB41L1  ERH  FGR  FN3K  FNBP1  FTCD  G6PD  GCLM  GLO1  GLRX3  GMPS  GPX1  GRK2  H1.10  H1.2  H2AC1  H2AC11  H2AW  H2BC12  H2BC21  H2BU1  HAGH  HARS1  HBEGF  HCLS1  HDAC2  HDGF  HEBP1  HMBS  HMGB2  HNRNPA0  HNRNPA1  HNRNPA2B1  HNRNPAB  HNRNPD  HNRNPK  HNRNPM  IFI16  IGBP1  IMPDH1  KARS1  KPNB1  LANCL2  LEAP2  LIMD2  LSP1  LY6G6C  MAGOH  MAP2K5  MAPKAPK3  MEP1A  METAP2  MFAP4  MNDA  MTHFD1  NACA  NANS  NAP1L1  NAPA  NCF1  NIT2  NME2  NMT1  NPW  NUDCD2  NUMB  OXSR1  PA2G4  PABPN1  PADI4  PAFAH1B3  PCBP2  PCNA  PCNP  PDCD5  PDE4C  PDXK  PDXP  PGR  PHPT1  PIN1  PIP4K2A  PIP4K2B  PKLR  PLEKHF2  PLPBP  PMM2  PNP  PPID  PPME1  PPP2R5A  PPP4R3A  PRDX1  PRDX3  PRKACA  PRKCB  PRPSAP2  PSIP1  PSMD11  PSMD9  PTPA  PTPN11  PURB  RAC2  RACK1  RAN  RANGAP1  RBM3  RECQL  RPL12  RPL26L1  RPL30  RPL5  RPS20  RPS25  RPS4X  RRM1  RTCA  RWDD1  RWDD4  S100A12  SFTPD  SH3GLB2  SH3PXD2B  SMC3  SNCA  SNRPB2  SNRPG  SNU13  SNX15  SPI1  SRGN  STAT1  STK24  STMN2  STMN4  TATDN1  TBCEL  TCEA1  TFF1  TIPRL  TMOD1  TMPO  TPD52L2  TTLL12  TTPAL  UBA1  UBA6  UBAC1  UBE2D1\|UBB  UBE2D3\|UBB  UBE2G1  UBE2Z  UBLCP1  USP14  USP15  VIM  WDR48  WFDC8  XRCC6  YBX1  YWHAH  YY1 | BPGM  C4A\|C4B  CCT5  CCT7  CHAC1  CLIC5  CST2  CST5  CTAG1A\| CTAG1B  EIF1B  GAA  GCG  HMBS  HMGCS1  HNRNPA0  INS  KLK14  KNG1  LANCL2  LEAP2  LEP  MGP  MLN  NGFR  NTS  PCBP2  PENK  PGD  PKLR  PLS3  PRL  PSMA7  PYY  TIMP3  TLR5  UBE2D1\| UBB  UBE2D3\| UBB | CHAC1  CST2  CST4  CST5  EPB41L1  FGA\|FGB\| FGG  HTN3  INS  LEP  MELT  PNLIP  UBE2D1\| UBB | ABHD14A  ABR  ADK  ADPRS  ADSL  AGO2  ANXA1  ANXA2  APBB1IP  APEX1  ARG1  BAGE2  BAP18  BPGM  BPI  CA1  CASP14  CCDC50  CCK  CDC42  CDH7  CFAP36  CHGA  CHMP1B  CHMP2B  CIAO1  CKB\|CKM  COQ9  CPNE1  CRYZ  CTAG1A\|CTAG1B  CTPS1  CXCL11  CXCL12  DDX39B  DDX6  DEF6  DEFA1  DENND10  DKK1  DKK4  ECHDC1  EEF1A1  EIF1B  ELMO2  ENO1  ERH  FGR  FN3K  FNBP1  FTCD  G6PD  GAA  GAPDH  GMPS  GRK2  H1.10  H1.2  H2AC1  H2AC11  H2AW  H2BC12  H2BC21  H2BU1  HBEGF  HK2  HMBS  HMGB2  HMGCS1  HNRNPA0  HNRNPA1  HNRNPA2B1  HNRNPAB  HNRNPD  HNRNPM  IFI16  IMPDH1  INPP5D  KARS1  KLC1  KLK7  KNG1  KPNB1  LANCL2  LAP3  LASP1  LDHA  LDLRAD4  LEAP2  LIMD2  LSP1  LY6G6C  MAGOH  MAPKAPK3  MARS1  MEP1A  MFAP4  MNDA  MPG  MTHFD1  MUL1  NANS  NCF1  NEK7  NIT2  NUDT16  PA2G4  PABPN1  PADI4  PAFAH1B3  PCBP2  PCDH8  PCNA  PDE4C  PDXK  PGD  PKLR  PKM  PLA2G2A  PLS3  PMM2  PNP  PPID  PRKCB  PSIP1  PSME2  RAB8B  RAC2  RACK1  RECQL  REN  RPL26L1  RPL30  RPL5  RPS25  RPS4X  RRM1  RTCA  S100A12  SAMSN1  SAR1A  SDCBP  SFTPD  SGTA  SH3GLB2  SMC3  SNRPA  SNRPB2  SNX27  SPI1  SRC  SRGN  STMN4  TBCEL  TCEA1  TFF1  TIMP3  TLR5  TPD52L2  TTC1  TTPAL  UBA1  UBE2D1\|UBB  UBE2D3\|UBB  UBLCP1  USP14  WFDC8  XRCC6  YARS1  YBX1  YY1  ZBP1 | ABR  ACAT1  ADPRS  AGR3  ALPI  ANXA1  APBB1IP  APEX1  ARG1  CDH7  CFAP36  CGGBP1  CHGA  CHMP1B  CHMP2B  COQ9  CSNK2A1\| CSNK2B  CXCL11  DDX39B  DEF6  DEFA1  DENND10  ECHDC1  ERH  FNBP1  FTCD  G6PD  GCG  GMPS  GPI  GRK2  H1.10  H1.2  H2AC1  H2AC11  H2AW  H2BC12  H2BC21  H2BU1  H3C1  HBEGF  HDGF  HK2  HMGB2  HNRNPA0  HNRNPA1  HNRNPA2B1  HNRNPD  HNRNPM  HSPA6  IFI16  IMPDH1  INPP5D  KARS1  KPNB1  LDHA  LEAP2  LEP  LIMD2  LSP1  LY6G6C  MAGOH  MAPKAPK3  MEP1A  MFAP4  MNDA  MPG  NANS  NCF1  PABPN1  PADI4  PCDH8  PDE4C  PDXK  PPP4R3A  PSIP1  RABL6  RECQL  RPL12  RPL26L1  RPL30  RPL5  RPS25  RPS4X  S100A12  SFTPD  SMC3  SNRPA  SNRPB2  SPI1  STMN4  TCEA1  TFF1  TPD52L2  UBE2D1\|UBB  UBE2G1  UBLCP1  VIM  WFDC8  XRCC6  YY1  ZBP1 |

**Supplementary Table S2A Common Upregulated Pathways Summary**

| **Vaccine received and timepoint** | **Male Recipients** | **Female Recipients** |
| --- | --- | --- |
| mRNA-1273  1-month | none | TLR signaling, fibrin clot formation, platelet aggregation, MAPK signalling |
| BNT162b2  1-month | APC-Cdc20 degradation, TICAM1and RIP-1 signaling, IKK complex recruitment | TICAM1 and RIP1 mediated signaling, IKK complex recruitment |
| mRNA-1273  6-months | Class-I antigen processing, metabolism of RNA, eucaryotic translation, peptide chain elongation | Metabolism of RNA, Peptide chain elongation, Neddylation, Class I Ag Processing |
| BNT162b2  6-months | Pathways associated with protein synthesis, cellular function including proliferation and apoptosis (SLIT-ROBO pathway), and suppression of interferon RIG-1 signaling (DDX58/IFIH1) | Innate Immune system activation, Glycolysis, Neddylation, Class I Ag Processing |

**Supplementary Table S2B Common Downregulated Pathways Summary**

| **Vaccine received and timepoint** | **Male Recipients** | **Female Recipients** |
| --- | --- | --- |
| mRNA-1273  1-month | none | Membrane trafficking, Vesicle trafficking, Endocytosis, Platelet Activation, RNA transport, IFN stimulated genes, Signaling: RAB, DAP12, MET, CD28, RTK, NTRKs, RET, IL-3, IL-5, GM-CSF, IFN |
| BNT162b2  1-month | Platelet activation, Antigen activation of B cell receptor, RNA transport, Membrane and RAB trafficking, Endosomal sorting complex formation (ESCRT), Endocytosis, Vesicle mediated transport, Platelet activation, Actin polymerization, Signaling: DAP12, EFGR, RTKs, MET | Membrane trafficking, Vesicle trafficking, Endocytosis, Platelet Activation, RNA transport, Signaling: RAB, DAP12, MET, CD28, RTK, NTRKs, RET, IL-3, IL-5, GM-CSF, IFN |
| mRNA-1273  6-months | Complement activation, Complement and coagulation cascades, Peptide ligand receptors | Membrane trafficking, Vesicle trafficking, Endocytosis, Platelet Activation, Signaling: RAB, DAP12, MET, CD28, RTK, NTRKs, RET, IL-3, IL-5, GM-CSF, IFN |
| BNT162b2  6-months | Membrane trafficking, Endocytosis, Rab regulation of trafficking, Signaling: EGFR, RTKs, MET, CD28, SCF-KIT, ESCRT | Protein synthesis, Membrane trafficking, Vesicle trafficking, Endocytosis, Platelet Activation. |

**Supplementary Table S3 Predictive Markers of 6-Month Serology**

| **Protein** | **Entrez Gene Symbol** | **Symbol** | **Average Higher Responder** | **Average Lower Responders** | **Change** | **Abundance in Higher Responders** | **Abundance in Lower Responders** |
| --- | --- | --- | --- | --- | --- | --- | --- |
| Dihydrolipoyl dehydrogenase, mitochondrial | P09622 | DLD | 1653.70897 | 1504.10568 | 1.09946329 | Higher | Lower |
| Signal transducer and activator of transcription 1-alpha/beta | P42224 | STAT1 | 407.898718 | 383.917045 | 1.06246577 | Higher | Lower |
| Unknown (SeqID.10461.57) | Unknown | Unknown | 313.074359 | 299.381818 | 1.04573605 | Higher | Lower |
| Anthrax toxin receptor 1 | Q9H6X2 | ANTXR1 | 2472.58846 | 2303.3 | 1.07349822 | Higher | Lower |
| Thrombospondin-type laminin G domain and EAR repeat-containing protein | Q8WU66 | TSPEAR | 1036.9359 | 1104.31932 | 0.93898194 | Lower | Higher |
| Oxidoreductase HTATIP2 | Q9BUP3 | HTATIP2 | 1336.09872 | 1210.25 | 1.10398572 | Higher | Lower |
| Thiopurine S-methyltransferase | P51580 | TPMT | 8164.7 | 7797.61023 | 1.04707721 | Higher | Lower |
| BPI fold-containing family B member 1 | Q8TDL5 | BPIFB1 | 27632.8795 | 20589.5886 | 1.34208021 | Higher | Lower |
| Tenascin-R | Q92752 | TNR | 2690.08205 | 2891.69773 | 0.93027775 | Lower | Higher |
| Serine/arginine-rich splicing factor 6 | Q13247 | SRSF6 | 403.275641 | 450.553409 | 0.89506734 | Lower | Higher |
| MICAL-like protein 2 | Q8IY33 | MICALL2 | 5029.05256 | 5317.07045 | 0.94583147 | Lower | Higher |
| cGMP-dependent protein kinase 1, beta isozyme | Q13976 | PRKG1 | 2839.81795 | 2757.08864 | 1.03000604 | Higher | Lower |
| Hydroxymethylglutaryl-CoA synthase, mitochondrial | P54868 | HMGCS2 | 617.697436 | 559.707955 | 1.10360668 | Higher | Lower |
| Carbonic anhydrase 6 | P23280 | CA6 | 6375.74359 | 5315.64545 | 1.1994298 | Higher | Lower |
| Ephrin-A4 | P52798 | EFNA4 | 2983.12308 | 3057.57727 | 0.97564928 | Lower | Higher |
| Membrane-associated guanylate kinase, WW and PDZ domain-containing protein 2 | Q86UL8 | MAGI2 | 612.015385 | 544.509091 | 1.12397643 | Higher | Lower |
| Carbonic anhydrase 4 | P22748 | CA4 | 1610.73333 | 1543.76818 | 1.04337772 | Higher | Lower |
| Protein S100-A4 | P26447 | S100A4 | 1877.10769 | 1672.71705 | 1.12219081 | Higher | Lower |
| Unknown (SeqID.14635.28) | Unknown | Unknown | 418.285897 | 399.993182 | 1.04573257 | Higher | Lower |
| Lysyl oxidase homolog 3 | P58215 | LOXL3 | 25359.1641 | 18870.358 | 1.34386238 | Higher | Lower |
| Neuregulin-1, sensory and motor neuron-derived factor isoform | Q02297 | NRG1 | 3413.44359 | 3073.92273 | 1.11045198 | Higher | Lower |
| Protein canopy homolog 4 | Q8N129 | CNPY4 | 4112.42308 | 3882.95455 | 1.05909637 | Higher | Lower |
| Alpha-N-acetylglucosaminidase | P54802 | NAGLU | 9406.55897 | 11488.9636 | 0.81874739 | Lower | Higher |
| Keratin, type I cytoskeletal 19 | P08727 | KRT19 | 3353.21154 | 3567.97045 | 0.93980922 | Lower | Higher |
| Ribonuclease T2 | O00584 | RNASET2 | 2367.93462 | 2108.88977 | 1.1228347 | Higher | Lower |
| Intestinal-type alkaline phosphatase | P09923 | ALPI | 3586.66154 | 2525.825 | 1.41999606 | Higher | Lower |
| Acyl-CoA synthetase family member 2, mitochondrial | Q96CM8 | ACSF2 | 1267.82821 | 1225.93977 | 1.03416843 | Higher | Lower |
| Krueppel-like factor 4 | O43474 | KLF4 | 682.335897 | 705.767045 | 0.96680045 | Lower | Higher |
| Clathrin light chain A | P09496 | CLTA | 1964.46538 | 1795.41023 | 1.09415963 | Higher | Lower |
| Kidney-associated antigen 1 | Q9UBP8 | KAAG1 | 5062.58846 | 4753.23182 | 1.06508343 | Higher | Lower |
| Carboxypeptidase A1 | P15085 | CPA1 | 3630.99615 | 3945.76136 | 0.920227 | Lower | Higher |
| 15 kDa selenoprotein | O60613 | SELENOF | 9057.86282 | 8124.20795 | 1.11492257 | Higher | Lower |
| Amphoterin-induced protein 1:Extracellular domain | Q86WK6 | AMIGO1 | 490.962821 | 470.080682 | 1.04442246 | Higher | Lower |
| Melanoma-associated antigen 3 | P43357 | MAGEA3 | 2173.77949 | 1953.63295 | 1.11268572 | Higher | Lower |
| Integrin alpha V beta 3 | P06756\|P05106 | ITGAV\|ITGB3 | 4907.28205 | 4235.62386 | 1.15857361 | Higher | Lower |
| DNA-directed RNA polymerases I, II, and III subunit RPABC2 | P61218 | POLR2F | 771.44359 | 693.785227 | 1.1119343 | Higher | Lower |
| Putative KHDC1-like protein | Q5JSQ8 | KHDC1L | 506.858974 | 473.423864 | 1.07062405 | Higher | Lower |
| Interleukin-17 receptor E | Q8NFR9 | IL17RE | 1499.24744 | 1641.87273 | 0.91313255 | Lower | Higher |
| Nectin-1, isoform gamma:Extracellular domain | Q15223 | NECTIN1 | 1757.23974 | 1653.1625 | 1.06295645 | Higher | Lower |
| Gliomedin | Q6ZMI3 | GLDN | 1993.65385 | 2235.56136 | 0.89179115 | Lower | Higher |
| Protein unc-119 homolog B | A6NIH7 | UNC119B | 1157.65 | 569.390909 | 2.03313748 | Higher | Lower |
| Charged multivesicular body protein 6 | Q96FZ7 | CHMP6 | 3624.61923 | 3488.28864 | 1.03908237 | Higher | Lower |
| Transmembrane and immunoglobulin domain-containing protein 2 | Q96BF3 | TMIGD2 | 5181.83462 | 4298.09773 | 1.20561117 | Higher | Lower |
| Retinol dehydrogenase 12 | Q96NR8 | RDH12 | 3580.24615 | 3358.89545 | 1.06589985 | Higher | Lower |
| ADAM 8 | P78325 | ADAM8 | 975.14359 | 868.979545 | 1.12217094 | Higher | Lower |
| Spermidine synthase | P19623 | SRM | 325.234615 | 276.036364 | 1.17823105 | Higher | Lower |
| Ubiquitin-related modifier 1 | Q9BTM9 | URM1 | 3233.22051 | 3457.85341 | 0.93503689 | Lower | Higher |
| OTU domain-containing protein 7B | Q6GQQ9 | OTUD7B | 247.983333 | 236.088636 | 1.05038234 | Higher | Lower |
| E3 ubiquitin-protein ligase CBL-C | Q9ULV8 | CBLC | 357.830769 | 325.331818 | 1.09989478 | Higher | Lower |
| HCE004331 | HCE004331 | HCE004331 | 120.75 | 119.088636 | 1.01395065 | Higher | Lower |
| Butyrophilin subfamily 3 member A3 | O00478 | BTN3A3 | 1533.76154 | 1613.77841 | 0.95041644 | Lower | Higher |
| C-C motif chemokine 7 | P80098 | CCL7 | 554.382051 | 524.077273 | 1.05782502 | Higher | Lower |
| HCE000483 | HCE000483 | HCE000483 | 203.4 | 201.019318 | 1.01184305 | Higher | Lower |
| Beta-dystroglycan | Q14118 | DAG1 | 3761.26154 | 3556.56818 | 1.05755362 | Higher | Lower |
| NIF3-like protein 1 | Q9GZT8 | NIF3L1 | 4286.08974 | 3721.70341 | 1.15164732 | Higher | Lower |
| Zinc finger protein 483 | Q8TF39 | ZNF483 | 2054.23462 | 1797.82614 | 1.1426214 | Higher | Lower |
| CDK-activating kinase assembly factor MAT1 | P51948 | MNAT1 | 2524.79359 | 2601.31364 | 0.97058408 | Lower | Higher |
| Citrate lyase subunit beta-like protein, mitochondrial | Q8N0X4 | CLYBL | 1316.20128 | 894.788636 | 1.47096334 | Higher | Lower |
| Laccase domain-containing protein 1 | Q8IV20 | LACC1 | 2678.9641 | 2770.24091 | 0.96705095 | Lower | Higher |
| SH3KBP1-binding protein 1 | Q8TBC3 | SHKBP1 | 1532.96667 | 3079.625 | 0.49777706 | Lower | Higher |
| GTPase IMAP family member 4 | Q9NUV9 | GIMAP4 | 1958.61923 | 1881.11136 | 1.04120323 | Higher | Lower |
| AP-1 complex subunit beta-1 | Q10567 | AP1B1 | 864.934615 | 824.914773 | 1.04851391 | Higher | Lower |
| Ferritin heavy polypeptide-like 17 | Q9BXU8 | FTHL17 | 2099.76282 | 1653.1375 | 1.27016828 | Higher | Lower |
| Methyltransferase-like 26 | Q96S19 | METTL26 | 1771.64103 | 1578.04545 | 1.12268061 | Higher | Lower |
| NT-3 growth factor receptor | Q16288 | NTRK3 | 6249.14103 | 5727.125 | 1.09114801 | Higher | Lower |
| Apolipoprotein A-I | P02647 | APOA1 | 18494.0808 | 16134.4534 | 1.14624774 | Higher | Lower |
| C-C motif chemokine 28 | Q9NRJ3 | CCL28 | 8370.37692 | 6778.04886 | 1.23492425 | Higher | Lower |
| Complement factor D | P00746 | CFD | 42197.7372 | 45807.6409 | 0.92119429 | Lower | Higher |
| Calpastatin | P20810 | CAST | 7114.27692 | 5775.71705 | 1.23175648 | Higher | Lower |
| Contactin-5 | O94779 | CNTN5 | 657.274359 | 633.689773 | 1.03721787 | Higher | Lower |
| E-selectin | P16581 | SELE | 27883.1231 | 24107.1375 | 1.15663351 | Higher | Lower |
| No protein |  |  | 3.16282051 | 2.71022727 | 1.16699457 | Higher | Lower |
| Legumain | Q99538 | LGMN | 4260.89744 | 3999.67955 | 1.0653097 | Higher | Lower |
| Granzyme B | P10144 | GZMB | 279.096154 | 270.344318 | 1.03237292 | Higher | Lower |
| Interleukin-20 | Q9NYY1 | IL20 | 363.592308 | 348.330682 | 1.04381361 | Higher | Lower |
| Pancreatic hormone | P01298 | PPY | 3042.45385 | 2325.70341 | 1.30818652 | Higher | Lower |
| Phospholipase A2 | P04054 | PLA2G1B | 675.011538 | 735.930682 | 0.91722163 | Lower | Higher |
| Reticulon-4 receptor | Q9BZR6 | RTN4R | 1219.47051 | 1313.57955 | 0.92835681 | Lower | Higher |
| Intercellular adhesion molecule 5 | Q9UMF0 | ICAM5 | 5456.73333 | 5958.14886 | 0.91584374 | Lower | Higher |
| 40S ribosomal protein S3a | P61247 | RPS3A | 1038.13077 | 998.788636 | 1.03938985 | Higher | Lower |
| Liver-expressed antimicrobial peptide 2 | Q969E1 | LEAP2 | 9616.84872 | 7162.72386 | 1.34262452 | Higher | Lower |
| Teratocarcinoma-derived growth factor 1 | P13385 | TDGF1 | 1288.14359 | 815.707955 | 1.57917252 | Higher | Lower |
| Leukemia inhibitory factor receptor | P42702 | LIFR | 2271.44615 | 2108.7 | 1.07717843 | Higher | Lower |
| T-lymphocyte activation antigen CD86 | P42081 | CD86 | 6014.53462 | 5603.97955 | 1.07326134 | Higher | Lower |
| von Willebrand factor A domain-containing protein 1 | Q6PCB0 | VWA1 | 664.365385 | 611.790909 | 1.08593537 | Higher | Lower |
| Cholecystokinin | P06307 | CCK | 1594.37308 | 1461.78864 | 1.09070014 | Higher | Lower |
| Matrilin-4 | O95460 | MATN4 | 1295.37308 | 1414.06477 | 0.91606347 | Lower | Higher |
| Synaptotagmin-7 | O43581 | SYT7 | 527.501282 | 555.726136 | 0.94921086 | Lower | Higher |
| Ribonuclease pancreatic | P07998 | RNASE1 | 345.735897 | 404.797727 | 0.85409545 | Lower | Higher |
| D-glucuronyl C5-epimerase | O94923 | GLCE | 3674.1 | 3409.84773 | 1.0774968 | Higher | Lower |
| Neurotensin/neuromedin N | P30990 | NTS | 2669.42308 | 1850.46023 | 1.44257252 | Higher | Lower |
| Transmembrane protein 132A | Q24JP5 | TMEM132A | 2748.48718 | 2486.55682 | 1.10533858 | Higher | Lower |
| Serine palmitoyltransferase 1 | O15269 | SPTLC1 | 4691.65256 | 5089.53409 | 0.92182359 | Lower | Higher |
| Protein FAM177A1 | Q8N128 | FAM177A1 | 3627.27949 | 3293.46818 | 1.10135556 | Higher | Lower |
| Mannan-binding lectin serine protease 1:Mannan-binding lectin serine protease 1 heavy chain | P48740 | MASP1 | 444.802564 | 403.65 | 1.10195111 | Higher | Lower |
| Intercellular adhesion molecule 5 | Q9UMF0 | ICAM5 | 2215.96667 | 2546.09432 | 0.87033958 | Lower | Higher |
| Peroxisomal membrane protein PEX14:N-term | O75381 | PEX14 | 464.562821 | 417.727273 | 1.11211992 | Higher | Lower |
| UPF0160 protein MYG1, mitochondrial | Q9HB07 | MYG1 | 2393.05 | 2610.61477 | 0.91666148 | Lower | Higher |
| Unknown (SeqID.8316.36) | Unknown | Unknown | 632.95 | 565.744318 | 1.11879162 | Higher | Lower |
| R-spondin-2 | Q6UXX9 | RSPO2 | 908.424359 | 975.193182 | 0.93153272 | Lower | Higher |
| RING finger protein 215:Cytoplasmic domain | Q9Y6U7 | RNF215 | 2872.23333 | 2784.46705 | 1.03151996 | Higher | Lower |
| Mucin-1:region 2 | P15941 | MUC1 | 1379.26923 | 1439.82727 | 0.95794076 | Lower | Higher |
| Lysosomal alpha-glucosidase | P10253 | GAA | 18553.2974 | 15816.0864 | 1.17306501 | Higher | Lower |
| Vesicular integral-membrane protein VIP36 | Q12907 | LMAN2 | 5043.53205 | 5506.69773 | 0.91589048 | Lower | Higher |
| MANSC domain-containing protein 4 | A6NHS7 | MANSC4 | 549.117949 | 608.960227 | 0.9017304 | Lower | Higher |
| RING finger protein 150 | Q9ULK6 | RNF150 | 440.721795 | 482.696591 | 0.91304103 | Lower | Higher |
| Catenin beta-1 | P35222 | CTNNB1 | 1386.6859 | 963.710227 | 1.43890337 | Higher | Lower |
